# Accuracy optimized neural networks do not effectively model optic flow tuning in brain area MSTd

**DOI:** 10.3389/fnins.2024.1441285

**Published:** 2024-09-02

**Authors:** Oliver W. Layton, Scott T. Steinmetz

**Affiliations:** ^1^Department of Computer Science, Colby College, Waterville, ME, United States; ^2^Center for Computing Research, Sandia National Labs, Albuquerque, NM, United States

**Keywords:** optic flow, deep learning, neural networks, sparse coding, MSTd, motion, dorsal stream, self-motion

## Abstract

Accuracy-optimized convolutional neural networks (CNNs) have emerged as highly effective models at predicting neural responses in brain areas along the primate ventral stream, but it is largely unknown whether they effectively model neurons in the complementary primate dorsal stream. We explored how well CNNs model the optic flow tuning properties of neurons in dorsal area MSTd and we compared our results with the Non-Negative Matrix Factorization (NNMF) model, which successfully models many tuning properties of MSTd neurons. To better understand the role of computational properties in the NNMF model that give rise to optic flow tuning that resembles that of MSTd neurons, we created additional CNN model variants that implement key NNMF constraints – non-negative weights and sparse coding of optic flow. While the CNNs and NNMF models both accurately estimate the observer's self-motion from purely translational or rotational optic flow, NNMF and the CNNs with nonnegative weights yield substantially less accurate estimates than the other CNNs when tested on more complex optic flow that combines observer translation and rotation. Despite its poor accuracy, NNMF gives rise to tuning properties that align more closely with those observed in primate MSTd than any of the accuracy-optimized CNNs. This work offers a step toward a deeper understanding of the computational properties and constraints that describe the optic flow tuning of primate area MSTd.

## Introduction

Since the introduction of AlexNet (Krizhevsky et al., 2012), convolutional neural networks (CNNs) have revolutionized the field of computer vision and have reached the point where they rival or exceed human performance on certain image recognition tasks (Ciresan et al., 2012; Lee et al., 2017; Phillips et al., 2018). Despite their recent impact on computer vision, CNNs have longstanding roots in visual neuroscience (Serre, 2019; Lindsay, 2021). Hubel and Wiesel proposed that the visual system is organized hierarchically based on their seminal discovery of simple and complex cells in cat visual cortex (Hubel and Wiesel, 1962; Hubel, 1982). Fukushima (1980) instantiated this theory as one of the first CNNs called Neocognitron. Remarkably, Neocognitron contains mechanisms that CNNs still use today, including the rectified linear unit activation function (ReLU) that models the nonnegativity and nonlinearity of neuronal firing rates and the max pooling operation that facilitates invariance in pattern recognition.

Given that CNNs contain biologically-inspired mechanisms, it is fascinating that these artificial neural networks have emerged as highly effective at predicting neural responses in areas along the primate ventral stream that include V1 (Cadena et al., 2019; Burg et al., 2021), V4 (Yamins et al., 2013; Guclu and Van Gerven, 2015), and IT (Khaligh-Razavi and Kriegeskorte, 2014; Yamins and Dicarlo, 2016). CNNs that explain most of the variance of neural responses in ventral stream areas generally achieve the highest image classifcation accuracy on the ImageNet dataset (Yamins et al., 2014; Schrimpf et al., 2018), a collection of more than one million natural images (Deng et al., 2009).

**Figure 1:**
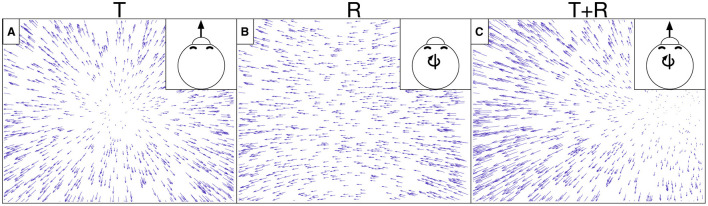
Example optic flow fields generated from simulated self-motion through a 3D dot cloud environment. Self-motion along a straight path of travel creates translational optic flow (T) with motion that radiates from the direction of movement. Eye or head movements yield rotational optic flow (R). **(A)** depicts translational optic flow from straight-forward self-motion. **(B)** depicts rotational optic flow corresponding to a rightward eye movement (yaw rotation). **(C)** depicts the optic flow created from a combination of translation and rotation (T+R). The optic field shown in **(C)** is the sum of those in **(A, B)**.

While such accuracy-optimized CNNs have had success in modeling neurons along the ventral stream, it is largely unknown whether they effectively model neurons in the complementary primate dorsal stream. Here we investigated the extent to which CNNs account for well-established tuning properties in dorsal stream area MSTd where neurons demonstrate selectivity to the expansive motion patterns experienced during self-motion (optic flow) (Duffy and Wurtz, 1995; Takahashi et al., 2007). MSTd neurons exhibit systematic tuning to combinations of translational (T) and rotational (R) optic flow (Graziano et al., 1994), which, to first-order approximation, capture any optic flow pattern (Figure 1). The translation and rotation directions that elicit the maximal response in individual neurons are not uniformly represented across the MSTd population—neurons are more likely to demonstrate a preference for optic flow corresponding to the lateral and vertical axes of self-motion (Takahashi et al., 2007). While this may seem counter-intuitive, MSTd neurons do nevertheless demonstrate increased discriminability of forward self-motion (Gu et al., 2010). Most MSTd neurons show a ≈90° difference in the translation and rotation directions that yield the maximal response (Takahashi et al., 2007). There is strong evidence that optic flow sensitivity in MSTd stems from the feedforward integration of local direction and speed signals from MT and other afferent visual areas (Maunsell and Van Essen, 1983; Perrone, 1992; Born and Bradley, 2005).

**Figure 2:**
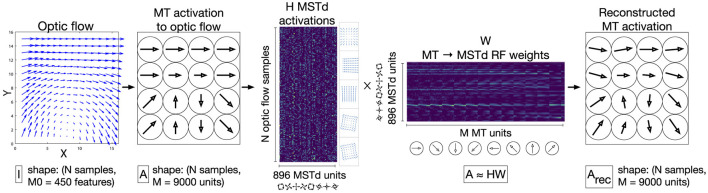
Schematic depiction the NNMF model of MSTd proposed by Beyeler et al. (2016). A population of *M* = 9, 000 speed and direction tuned MT units integrate each input 15 × 15 optic flow field, generating a *N*×9, 000 matrix of activations (*A*), where *N* denotes the number of optic flow samples **(left two panels)**. This matrix serves as the input to the NNMF algorithm, which factors *A* into the matrices *H* and *W* (center panels). *H* corresponds to the nonnegative basis coefficients, which is interpreted as the activations of the 896 model MSTd units to the *N* optic flow samples. *W* corresponds to 896 basis vectors, which is interpreted as the MSTd RF connection weights from MT units. The product *A*_*rec*_ = *HW* is a reconstruction of the MT input activations *A* **(right panel)**.

Beyeler et al. (2016) introduced the Non-Negative Matrix Factorization (NNMF) model of MSTd that reproduces the aforementioned optic flow tuning properties as well as others that will be described later on in this article. NNMF refers to the process of approximating a matrix *A* with the product of two other matrices *H* and *W*, where all three matrices have nonnegative entries. This forms a basis with which the original matrix may be reconstructed, and, similar to principal component analysis (PCA), the basis has fewer dimensions than the original matrix. Beyeler et al. (2016) apply NNMF to the local motion responses of model MT units to optic flow (*A*) and interpret the resulting *W* matrix as MT-MSTd receptive field (RF) connection weights (basis vectors) and *H* matrix as MSTd unit activations (basis coefficients). This NNMF model can be thought of as a two-layer neural network that, once fit, represents a simple linear model of MT activations (Figure 2). Unlike PCA, NNMF yields a sparse “parts-based” representation of optic flow patterns wherein many fitted coefficients equal zero (Lee and Seung, 1999; Beyeler et al., 2019).

In the present study, we optimized CNNs to estimate the translational and rotational self-motion from each optic flow field in a large dataset. We compared the tuning characteristics of neurons in these CNNs to both neurophysiological studies of MSTd (Ben Hamed et al., 2003; Takahashi et al., 2007) and tuning characteristics of the NNMF model proposed (Beyeler et al., 2016). To better understand what makes NNMF a successful model of MSTd, we trained CNN variants that incorporate one or both key computational properties of NNMF: nonnegative weights and sparse coding.

## Materials and methods

We begin by describing the optic flow datasets used to train and evaluate the computational models. We subsequently present the model specifications and end the section with a description of our analyses. To facilitate comparisons with the NNMF model of MSTd, many of our datasets and analyses are based on those of Beyeler et al. (2016), which replicate a number of analyses performed in previous primate studies within a computational modeling framework. These analyses focus on neural tuning to translational and rotational optic flow and encompass direction preference, tuning width, degree of selectivity, and sparseness of the neural signal.

### Optic flow datasets

**Figure 3:**
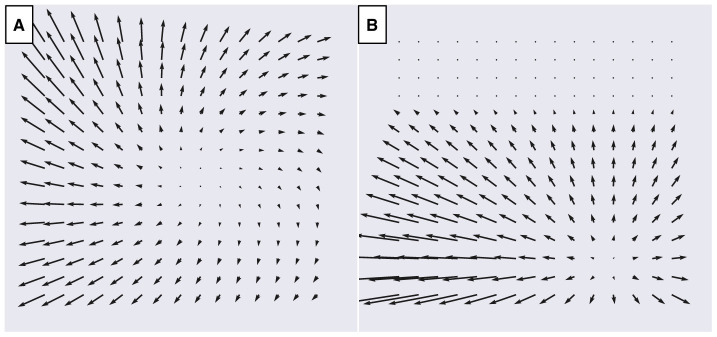
Examples of optic flow fields from the TR360 dataset used to fit the models. **(A)** Simulated translation and rotation with respect to a frontoparallel plane 4 m in front of the observer. Translation is -29° in azimuth (x) and 7° in elevation (y) at 1 m/s. The simulated observer undergoes 10°/sec rotation (3D rotation unit vector $\vec{R}=\left[0.33,0.79,0.52\right]$). **(B)** Simulated translation with respect to a ground plane with a 30° downward gaze offset (α = −30°). Translation is 28° in azimuth (x) and -33° in elevation (y) at 1.5 m/s.

Our datasets consist of the optic flow generated during simulated self-motion through visual scenes that comprise either a frontoparallel plane (Figure 3A) or ground plane (Figure 3B). Each data sample corresponds to a single vector field that represents the optic flow produced during an instant of time when the observer moves with 3D translational velocity $\vec{T}=\left(T_{x},T_{y},T_{z}\right)$ and rotational velocity $\vec{R}=\left(R_{x},R_{y},R_{z}\right)$. We simulated an observer with a 90° field of view and assume that the projection of points from the visual scene onto the retinal image plane occurs using a pinhole camera model with focal length *f* = 1 cm (Raudies and Neumann, 2013). Following Beyeler et al. (2016), we compute the optic flow on an evenly spaced 15 × 15 grid of points within retinal image plane. The *x* and *y* coordinates of these points both span [−*f, f*], the central portion of the image plane that falls within the simulated 90° field of view (Raudies and Neumann, 2013; Beyeler et al., 2016). Given a translation $\left(\vec{T}\right)$ vector, a rotation vector $\left(\vec{R}\right)$, and the 2D grid of sample points on the image plane (*x, y*), we evaluated the instantaneous optic flow (ẋ, ẏ) (Longuet-Higgins and Prazdny, 1980):


(1) $$\begin{array}{l} \;\left(\begin{array}{c} \dot{x}\\ \dot{y} \end{array}\right)=\frac{1}{Z(x,y)}\left(\begin{array}{ccc} -f & 0 & x\\ 0 & -f & y \end{array}\right)\left(\begin{array}{c} T_{x}\\ T_{y}\\ T_{z} \end{array}\right)\\ +\frac{1}{f}\left(\begin{array}{ccc} xy & -\left(f^{2}+x^{2}\right) & fy\\ \left(f^{2}+y^{2}\right) & -xy & -fx \end{array}\right)\left(\begin{array}{c} R_{x}\\ R_{y}\\ R_{z} \end{array}\right) \end{array}$$

In Equation 1, *Z*(*x, y*) refers to the depth of the point in the world that projects to (*x, y*) on the image plane. For the frontoparallel plane scene, *Z*(*x, y*) is set to the relative depth of the plane (see Table 1). For the ground plane scene, *Z*(*x, y*) = *Z*(*y*) = *hf*/(*ycos*(α)+*fsin*(α)), where *h* is the height of the ground plane relative to the eye, *f* is the camera focal length, and α is the vertical offset angle of the gaze relative to the horizontal axis. Following Beyeler et al. (2016), we set *h* = −10 m (i.e., ground plane is 10 m below the observer) and α = −30° (i.e., observer gaze is directed 30° below the horizon). The shape of each optic flow dataset is (*N*, 15, 15, 2), where *N* indicates the number of data samples and 2 corresponds to the (ẋ, ẏ) optic flow components.

**Table 1:** Optic flow dataset specifications.

**Dataset**	**Description**	**Size (Num samples N)**	**Independent variables**
TR360	Simulated self-motion toward either a frontoparallel plane or above a ground plane. T and R direction is uniform random: T/R elevation [–180, 180]°, T/R azimuth [–90, 90]°.	total: 12,060 (6,030 frontoparallel + 6,030 ground) train: 6030 validation: 3,015 test: 3015	T speed: [0.5, 1.0, 1.5] m/s R speed: [0, 5, 10] m/s Frontoparallel plane depth: [2, 4, 8, 16, 32] m
BenHamedT	Simulated self-motion (T only) toward a frontoparallel plane. Direction of azimuth and elevation is uniform random within [–45, 45]° of straight-ahead. Observer speed is uniform random within [0.5, 2]°/s.	10,000	Frontoparallel plane depth: [1, 2, 4, 8] m
BenHamedR	Simulated self-motion (R only) with a frontoparallel plane. Combinations of pitch and yaw rotation only, no roll. Net rotational speed is uniform random within [0, ±10] °/s.	10,000	Frontoparallel plane depth: [1, 2, 4, 8] m
TestProtocolT	Diagnostic set of optic flow patterns used to evaluate MSTd tuning to specific T directions.	514 (512 combinations of T azimuth and elevation & ±90° vertical)	T azimuth: [0, ±11.25, ±22.5, ..., ±180]° T elevation: [0, ±11.25, ±22.5, ..., ±90]°
TestProtocolR	Diagnostic set of optic flow patterns used to evaluate MSTd tuning to specific R directions.	514 (512 combinations of T azimuth and elevation & ±90° vertical)	R azimuth: [0, ±11.25, ±22.5, ..., ±180]° R elevation: [0, ±11.25, ±22.5, ..., ±90]°

T and R denote translation and rotation, respectively. T indicates optic flow that contains only translation, T/R indicates optic flow with both translational and rotational components. Training set is used to fit network weights, while test set is used to evaluate performance on novel patterns. Uniform indicates sampling from the uniform random distribution with the specified endpoints. Azimuth and elevation angles of 0° correspond to straight-ahead heading. See text for details.

Table 1 summarizes the datasets used in the present study. Each one adheres closely to the specifications provided by Beyeler et al. (2016). We fit each of the models with the TR360 training set and the remaining datasets serve as test sets to evaluate performance on optic flow that is not used to fit the models (i.e., to test generalization). To create the reported total number of samples, unless noted otherwise, we crossed the independent variables with each other then generated repetitions of these conditions until the number of samples matched the total number. For example, to generate the 6,030 TR360 samples for the frontoparallel scene, we crossed T speed (3), R speed (3), and frontoparallel plane depth (5) to obtain 45 samples. We subsequently repeated this process 134 times to obtain the 6,030 total fronotoparallel samples. We drew values for indicated random variables (T and R directions in this case) anew when generating each sample so that the dataset does not contain duplicate samples.

In the TR360 dataset, half of samples correspond to simulated self-motion toward the frontoparallel plane and the other half correspond to simulated self-motion over the ground plane. We shuffled the order of samples before fitting the models and creating the train/validation/test set splits.

### Computational models

#### Convolutional neural networks

**Figure 4:**
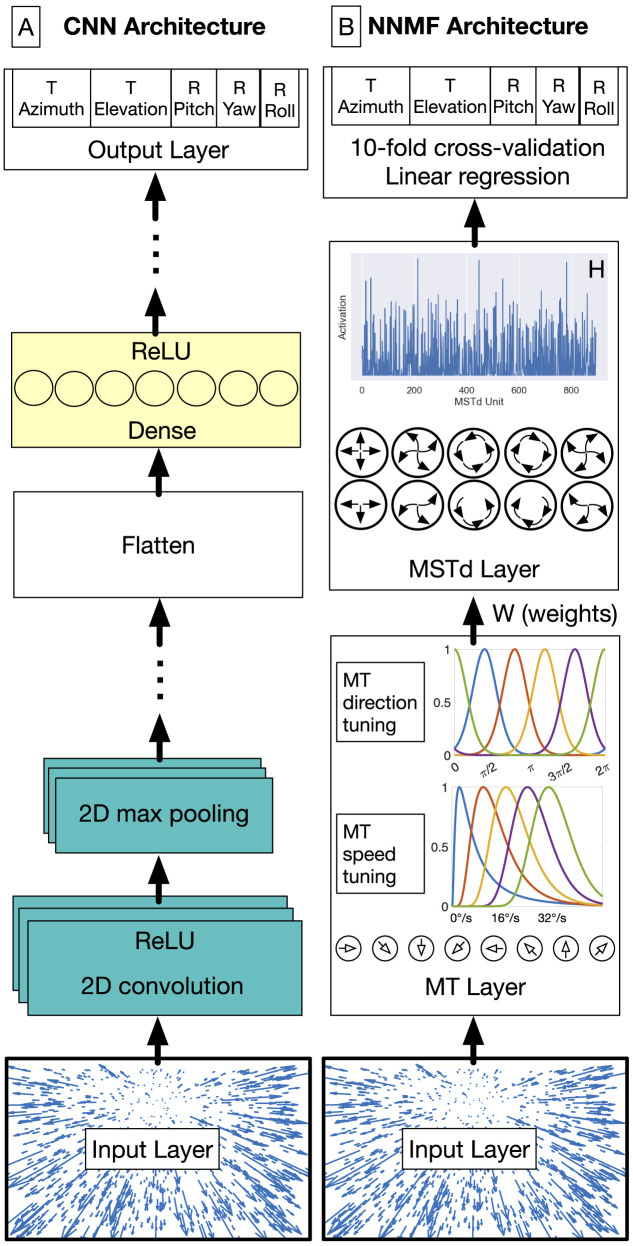
CNN **(A)** and NNMF **(B)** model diagrams. **(A)** The first stages of the CNN consist of convolutional units that filter the optic flow field within the RF. This is followed by the max pooling operation, which downsamples the spatial resolution of the optic flow signal. Convolution and max pooling layers may be stacked multiple times before the units are flattened into a 1D representation. These units are connected with one or more layers of densely connected units. The output layer consists of five neurons, one for each self-motion property that the network estimates (the azimuth and elevation of observer translation as well as the pitch, yaw, and roll components of observer rotation). The network minimizes the mean-squared-error (MSE) loss for regression. The MLP model excludes the convolution and max pooling stages (teal). **(B)** In the NNMF model, MT units that are preferentially tuned to a unique combination among 8 directions and 5 speeds integrate optic flow within their RF. MSTd activations emerge by multiplying the MT activations with the MT–MSTd weights obtained from the NNMF algorithm.

We implemented the CNNs using TensorFlow 2.11 in Python 3.10 (Abadi et al., 2016). We trained the models on a Microsoft Windows 10 workstation equipped with an NVIDIA GeForce RTX 4090 graphics processing unit (GPU) that has 24 GB of RAM. As depicted in Figure 4A, the general CNN architecture processes the optic flow at the input layer with shape (*N*, 15, 15, 2). The CNN applies 2D spatial convolution to the input signal with stride 1 and zero padded with “same” boundary conditions. This is followed by the ReLU activation function and the 2D max pooling operation. The 2D convolution and max pooling layers are stacked together one or more times. The units in the final max pooling are flattened and connected to one or more layers with dense connectivity and the ReLU activation function. The final stage is a multi-output regression layer with 5 neurons, each representing the self-motion variables that we train the network to estimate: translational azimuth and elevation, as well as rotational pitch, yaw, and roll. Prior to training, we normalized each label separately so that each spans the range [–0.5, 0.5]. The goal of the training process is to minimize the loss incurred in jointly estimating the 5 self-motion variables using the Adam optimizer, configured with default hyperparameters except as noted below. We use mean squared error (MSE) loss for translational elevation (unscaled range: [–90, 90]°) and the rotation variables (unscaled range: [–10, 10]°/s). For the translational azimuth, the network minimizes the following loss function since the range is circular [–180, 180]°:


(2) $$\begin{array}{l} L_{circ}=\frac{1}{B}\overset{B}{\underset{i=1}{\sum }}\frac{1}{2}\left[1-\cos\left(\pi \left(y_{i}-{ŷ}_{i}\right)\right)\right] \end{array}$$

In Equation 2 *B* is the mini-batch size, *y*_*i*_ is the translational azimuth label for sample *i* on the normalized scale, and ŷ_*i*_ is the normalized predicted translational azimuth value on the normalized scale. This loss function yields the maximum penalty of 1 when the error is 180° offset from the label (e.g., predicting straight-ahead when optic flow of moving straight-back) and 0 penalty when the error is zero or 360° offset from the label. That is, this loss function does not penalize learning angles that deviate by multiples of 360°.

We considered the extent to which several CNN variants modeled MSTd tuning properties:

1. MLP. Multi-Layer Perceptron that possessed the same architecture as the CNN shown in Figure 4A, but without the convolution and max pooling layers (without the teal boxes).
2. MLP_MT_PRE. Identical to MLP except the input to the neural network is the same as that of the NNMF model—activations obtained from the layer of 9000 model speed and direction tuned MT neurons rather than optic flow.
3. CNN_L1. The CNN with L1 regularization added to the loss and weights in each layer. The regularization strength was controlled independently in each layer and the values were determined through the optimization process described below.
4. CNN_++. The CNN optimized subject to the constraint that the weights, except for those in the first hidden layer and the output layer, must be non-negative.
5. CNN_L1++. The CNN optimized with both L1 regularization and the non-negative weight constraint.

We initialized the weights in each network using the Glorot Uniform method, the TensorFlow default (Glorot and Bengio, 2010; Abadi et al., 2016). This means that the initial value of each weight is drawn from the following Uniform distribution:


(3) $$\begin{array}{l} U\left[-\sqrt{\frac{6}{F_{in}+F_{out}}},\sqrt{\frac{6}{F_{in}+F_{out}}}\right] \end{array}$$

In Equation 3, *F*_*in*_ and *F*_*out*_ refer to the number of units in the previous and current layer, respectively. We initialized the weights of the networks with the non-negative weight constraint according to the absolute value of Equation 3 to ensure that the weights would initially take on nonzero values.

We optimized the neural network architecture and hyperparameters through a two-stage random search process. During the first stage, we searched for the CNN network hyperparameters listed in Table 2. This involved fitting the CNN model to the TR360 training set and recording the hyperparameters that yielded the smallest validation loss summed across the 5 output neurons. Whenever the search identified an optimal CNN candidate that possessed a hyperparameter value that encroached on the upper limit of its respective range, we expanded the range and restarted the search. During the second stage, we ran independent searches to optimize each of the variant networks. We conducted the searchs over the hyperparameter ranges in Table 2, which are informed by the first stage. We trained all networks with a mini-batch size of 64 samples and early stopping with a patience of 60 epochs (see Supplementary Figure S1 for model training and validation loss curves). Table 3 shows the optimized hyperparameters values for each of the networks.

**Table 2:** Ranges used in random search for optimal CNN hyperparameters.

**Hyperparameter**	**Value range**
Number of convolution and max pooling stacks	[1, 3]
Number of dense layers	[1, 6]
Number of convolutional filters	[2, 300]
Number of dense units	[2, 10,000]
Convolutional unit filter size	[2, 15]
Max pooling window size	[2, 4]
Max pooling stride length	[1, 3]
L1 regularization strength	[0, 1e-5, 1e-4, 1e-3, 1e-2, 1e-1, 1, 2]^*^
Learning rate	[1e-5, 1e-4, 1e-3, 1e-2]^**^

Except for the learning rate, hyperparameters were selected on a per-layer basis on every iteration of the search. ^*^L1 regularization was only included in CNN_L1 and CNN_L1++ networks and was drawn randomly from this set. ^**^Learning rate was drawn randomly from this set.

**Table 3:** Optimized network hyperparameters.

**Hyperparameter**	**CNN**	**MLP**	**MLP_MT_PRE**	**CNN_L1**	**CNN_++**	**CNN_L1++**
Number of convolution and max pooling stacks	1	0	0	1	1	1
Number of dense layers	5	5	5	3	2	2
Number of convolutional filters	157	0	0	188	162	103
Number of dense units	[2,997, 7,566, 5,979, 6,709, 2,631]	[2,958, 6,244, 3,234, 5,067, 2,651]	[3,536, 2,367, 141, 5,108, 408]	[5,484, 7,352, 9,770]	[3,145, 2,529]	[7,281, 5,741]
Convolutional unit filter size	2	N/A	N/A	2	2	2
Max pooling window size	2	N/A	N/A	4	2	2
Max pooling stride length	3	N/A	N/A	1	1	2
L1 regularization strength	0	0	0	[1e-5, 0, 1e-5, 1e-3, 0]	0	[0, 1e-4, 1e-5, 1e-3]
Learning rate	1e-3	1e-3	1e-3	1e-4	1e-3	1e-3

Entries in lists correspond to the values in each relevant layer of the network. For example, [3,145, 2,529] means that there are 3,145 units in the first dense hidden layer and 2,529 units in the second dense hidden layer.

#### Non-negative matrix factorization model

We implemented and configured the NNMF model according to the specifications of Beyeler et al. (2016), except where noted otherwise.

The first stage of the model (Figure 4B) transforms each 15 × 15 optic flow field into motion signals in model area MT. Model MT neurons exhibit sensitivity to the speed and direction of optic flow signals over time. We simulated 9000 (*N*_*MT*_) model MT neurons: 8 preferred directions and 5 preferred speeds centered on each of the 15 × 15 positions in the optic flow input (9, 000 = 8 × 5 × 15 × 15).

Tuning to the direction θ(*x, y*) present at position (*x, y*) within the RF of a neuron relative to the preferred direction θ_*pref*_ obeys a von Mises distribution (Equation 4):


(4) $$\begin{array}{l} d_{MT}\left(x,y;\theta_{pref}\right)=exp\left(\sigma_{\theta }\left(cos\left(\theta \left(x,y\right)-\theta_{pref}\right)-1\right)\right) \end{array}$$

where σ_θ_ = 3° indicates the bandwidth of the direction tuning, which was set to approximate the ≈90° full-width at half-maximum found in MT cells (Britten and Van Wezel, 1998; Beyeler et al., 2016).

The tuning of each model MT neuron to the optic flow speed (ν) at position (*x, y*) within the RF obeys a log-normal distribution (Nover et al., 2005):


(5) $$\begin{array}{l} s_{MT}\left(x,y;\nu_{pref}\right)=exp\left(-\frac{log{\left(\frac{\nu \left(x,y\right)+s_{0}}{\nu_{pref}+s_{0}}\right)}^{2}}{2\sigma_{\nu }^{2}}\right) \end{array}$$

where σ_ν_ in Equation 5 defines the speed tuning bandwidth, *s*_0_ defines a non-negative offset parameter to prevent the singularity in the logarithm at 0, and ν_*pref*_ defines the preferred speed of the model neuron. We set σ_ν_ = 1.16 and *s*_0_ = 0.33 to match the median values obtained from neural data (Nover et al., 2005). The 5 model MT preferred speeds are 2, 4, 8, 16, and 32°/s (Nover et al., 2005; Beyeler et al., 2016).

The activation of each model MT neuron is the product of the direction and speed inputs within the RF (Equation 6):


(6) $$\begin{array}{l} A=d_{MT}\left(x,y;\theta_{pref}\right)s_{MT}\left(x,y;\nu_{pref}\right) \end{array}$$

We used NNMF to decompose the MT activation matrix *A* into the product of two other matrices *HW*, all of which must have non-negative entries:


(7) $$\begin{array}{l} A\approx HW \end{array}$$

The MT matrix *A* has shape (*N, M*), where *N* corresponds to the number of optic flow samples and *M* corresponds to the number of MT units. The matrix *H* has shape (*N, K*) and represents the activations of the *K* = 896 model MSTd units to each sample. The matrix *W* has shape (*K, M*) represents the weights between the *M* = 9, 000 MT units and the *K* = 896 model MSTd units. NNMF minimizes the MSE reconstruction loss *L*_*nnmf*_ between *A*_*rec*_ = *HW* (Equation 7) and *A*


(8) $$\begin{array}{l} L_{nnmf}=\frac{1}{NM}\overset{N}{\underset{i=1}{\sum }}\overset{M}{\underset{j=1}{\sum }}{\left(A_{ij}-A_{rec_{ij}}\right)}^{2}, \end{array}$$

subject to the non-negativity constraint that all entries *H*_*ik*_≥0 and *W*_*kj*_≥0. We implemented NNMF in TensorFlow and used gradient descent with the Adam optimizer (learning rate: 1e-3) to minimize *L*_*nnmf*_ (Equation 8). We enforced the non-negativity constraint on each training epoch through the ReLU function.

NNMF has several free hyperparameters that must be specified. First, the matrices *H* and *W* require initialization. We used uniform random values between 0 and $\sqrt{\frac{\overset{N}{\underset{i=1}{\sum }}\overset{M}{\underset{j=1}{\sum }}A}{NMK}}$, as used in the Scikit-learn library implementation of NNMF (Pedregosa et al., 2011). Second, the number of basis vectors in the decomposition *K* must be selected. Following Beyeler et al. (2016), we repeatedly fit NNMF 14 times, using $\hat{K}=64$ basis vectors in each instance. This allows the model to incorporate variability in the fitted basis vectors due to the random initiation of *H* and *W*. We concatenated the matrices fit from each of the 14 NNMF fits to obtain *K* = 896. Third, NNMF requires convergence criteria for the iterative optimization of Equation 8 to be set. This is important since fitting NNMF for too many iterations risks overfitting the training set. We stopped the fit when the absolute difference in the loss obtained between successive training epochs was at most 1e-4. This is the default tolerance value used in the MATLAB function *nnmf*, which Beyeler et al. (2016) used to fit their model (we are assuming they used default parameter values since they do not state otherwise). We required NNMF to be fit to the training set for at least 2 epochs. Fourth, an algorithm must be selected by which NNMF is fit. We used gradient descent since it allowed us to fit NNMF using the same paradigm as the neural networks and our TensorFlow implementation allowed us to train quickly on the GPU. We obtained similar results using the alternating least-squares algorithm, which is what Beyeler et al. (2016) used.

We used the following equation (Equation 9) to compute MSTd activations *H*_*test*_ in the fitted NNMF to *N*_*test*_ novel stimuli.


(9) $$\begin{array}{l} H_{test}=A_{test}W^{T}, \end{array}$$

where *A*_*test*_ is the MT activations to the novel stimuli (shape: (*N*_*test*_, *M*)) and *W*^*T*^ is the transpose of the MT–MSTd fitted NNMF basis vectors (shape: (*M, K*)).

### Analyses

We focused our analyses of the units in the final hidden layer of the CNNs and the MSTd layer of the NNMF algorithm (*H*_*test*_). Unless noted otherwise, we excluded neurons that did not produce nonzero activation to any of the test optic flow patterns (“unresponsive neurons”/“dying ReLUs”) from the analysis (Glorot et al., 2011; Lu et al., 2019). Our analyses focus on model outputs to test sets, which represent novel optic flow that was not used to fit the models.

#### MSTd receptive field maps

We used using the TestProtocolT and TestProtocolR diagnostic datasets to characterize the tuning of model units to different translation and rotation directions. First, we interpolated the unit's activations obtained to the *N* = 514 optic flow patterns within each diagnostic dataset on a regular azimuth-elevation mesh. We subsequently evaluated the interpolation at 561 sample points made up of 33 azimuths (0–360° in 11.25° increments) and 17 elevations (–90–−90° in 11.25° increments). Finally, we created a heat map showing a Lambert cylindrical equal-area projection: the horizontal axis corresponds to the azimuthal angle θ, the vertical axis corresponds to the elevation angle ψ transformed as *sin*(ψ), and the color corresponds to the unit activation.

In addition to generating heat map plots showing individual unit tuning, we created composite heat maps showing the activation at each sample point averaged across the entire model population.

#### Translation and rotation tuning preferences

We established the translation and rotation tuning preferences of each unit using the TestProtocolT and TestProtocolR datasets (see Table 1). We estimated each neuron's preferred tuning using the population vector method (Georgopoulos et al., 1986; Beyeler et al., 2016). This involves summing the product between each unit activation and the corresponding optic flow translation or rotation labels (${\vec{T}}_{i}$ or ${\vec{R}}_{i}$), represented as 3D Cartesian unit vectors.

#### Population tuning width

To characterize the T or R tuning width of each model unit, we considered the interpolation obtained on the TestProtocolT and TestProtocolR datasets. We evaluated the interpolation at 100 azimuths and 50 elevations (3.6° increments along either axis). We defined a unit's tuning width as the Euclidean distance between where unit achieves its maximum and half-maximum activation.

#### Peak heading discriminability

To quantify how well individual model units discriminate between similar headings, we presented each model with 24 optic flow patterns with equally spaced translation directions spanning 0–360° (step size: 15°) in the horizontal plane (0° elevation). We fit separate cubic splines with 1000 equally spaced sample points to the set of activations produced by each model unit. We evaluated the first derivative of the spline to measure the discriminability at different reference directions of translation. Following Gu et al. (2006) and Beyeler et al. (2016), we report the peak heading discriminability, defined as the heading direction at which the spline derivative reaches its maximum. To mitigate edge and circularity effects, we padded the endpoints with activations produced to additional optic flow stimuli with 20 translation direction steps reaching ±300° on either side of the 0–360° central interval.

#### Heading and rotation tuning index

The translation direction (heading) that generates the strongest response represents the heading preference for a particular model unit. The heading tuning index (HTI) measures the selectivity of each unit's heading tuning. A neuron with a strong heading preference (HTI ≈ 1) activates only to a narrow range of heading directions, while a neuron that activates to a broad range of headings exhibits a weak preference (HTI ≈ 0).

We computed the HTI for each model unit *j* on the TestProtocolT dataset according to (Gu et al., 2006; Beyeler et al., 2016):


(10) $$\begin{array}{l} HTI_{j}=\frac{|\sum_{i=1}^{N}r_{ij}{\vec{e}}_{i}{|}_{2}}{\sum_{i=1}^{N}|r_{ij}{\vec{e}}_{i}{|}_{2}} \end{array}$$

where *r*_*ij*_ denotes the activation of the *j*^th^ model unit in response to the *i*^th^ stimulus, ${\vec{e}}_{i}$ denotes the heading direction for the *i*^th^ stimulus as a 3D Cartesian unit vector, and |·|_2_ denotes the *L*^2^ Euclidean norm. This equation produces HTI values ranging from 0 (no directional tuning) to 1 (strong directional tuning).

Moreover, we used using Equation 10 to compute a tuning index that measures each neuron's rotation selectivity that we refer to as the rotation tuning index (RTI). We computed the RTI of each neuron based on the activations obtained on the rotation-only TestProtocolR diagnostic dataset, substituting the 3D Cartesian rotation unit vector (${\vec{e}}_{i}$) for sample *i* in Equation 10.

#### Sparseness metrics

We assessed the sparseness (s) of model MT unit activity (r) using the following metric (Vinje and Gallant, 2000):


(11) $$s=\left(1-\frac{1}{N}\frac{{\left(\sum_{i=1}^{N}r_{i}\right)}^{2}}{\sum_{i=1}^{N}r_{i}^{2}}\right)/\left(1-\frac{1}{N}\right)$$

where *s* in Equation 11 is a metric ranging from 0 to 1, with 0 indicating a dense code where every neuron is always active and 1 indicating a local code where a single neuron is active for each stimulus. A sparse coding represents a middle ground, where a subset of neurons activate to any given stimulus. We use this equation to calculate two measures of sparseness. The first is population sparseness, the proportion of model units active for a single stimulus, where *r*_*i*_ represents the activity of the *i*^th^ model unit to a stimulus and *N* is the total number of model units. The second is lifetime sparseness, the proportion of stimuli a single model unit is active for, where *r*_*i*_ represents the activity of a model unit to the *i*^th^ stimulus and *N* is the total number of stimuli. The population and lifetime sparseness metrics reflect average values for all stimuli and model units, respectively.

### Software accessibility

We implemented and simulated NNMF and the CNNs in Python using the NumPy (Harris et al., 2020), SciPy (Virtanen et al., 2020), Pandas (McKinney, 2010), Seaborn (Waskom, 2021), and TensorFlow (Abadi et al., 2016) libraries. The trained neural network models and optic flow datasets are available on Hugging Face (https://huggingface.co/collections/OWLab/optic-flow-cnns-15x15-66bce4ab1c1fda3179e21b0c). Code to load the models and datasets is available on GitHub (https://github.com/owlayton/DL-MSTd-Acc-SelfMotion-Release).

## Results

Inspired by the success of accuracy-optimized CNNs at modeling neural activity in primate ventral stream areas, we examined the extent to which CNNs capture optic flow tuning in dorsal stream area MSTd. We optimized CNNs to estimate the observer's visual translation and rotation on a 6030 sample optic flow dataset (TR360) composed of 3D linear translation sampled from all possible directions and 3D rotation sampled from a range of commonly encountered speeds (0–10°/s) (see Table 1). In addition to a “baseline” CNN model (see Section Materials and methods), we simulated CNN variants that implement two key characteristics of the NNMF model of Beyeler and colleagues whose properties closely emulate many well-established characteristics of MSTd: sparse coding of optic flow and non-negativity in connectivity weights that gate afferent signals. Our aim was to examine how these computational principles influence the correspondence with MSTd properties. The three CNN variants that we considered are:

1. CNN with L1 regularization (LASSO) in each layer (Tibshirani, 1996), which promotes spareness in each neuron's weights (CNN_L1).
2. CNN optimized with non-negative weight constraints imposed on each internal network layer (CNN_++).
3. CNN with both L1 regularization and non-negative weight constraints (CNN_L1++).

To better understand how the convolution operation influences the emergence of MSTd-like optic flow tuning properties when the goal is accurate self-motion estimation, we compared the CNNs to multi-layer perceptions, which possess the same architecture except that they lack the convolution and max pooling layers early in the network. We considered two variants to gauge how training neural networks on a MT-like motion representation instead of optic flow may impact optic flow tuning. One multi-layer perception model (MLP) integrates the optic flow signal directly similar to how the CNNs do, whereas the other model (MLP_MT_PRE) integrates the responses of MT-like speed and direction tuned units to optic flow like NNMF does (see Materials and Methods and “MT Layer” panel of Figure 4B).

We begin by presenting the accuracy with which CNNs perform this task before comparing CNN translation and rotation tuning properties with those of primate area MSTd.

### Accuracy of self-motion estimates

**Figure 5:**
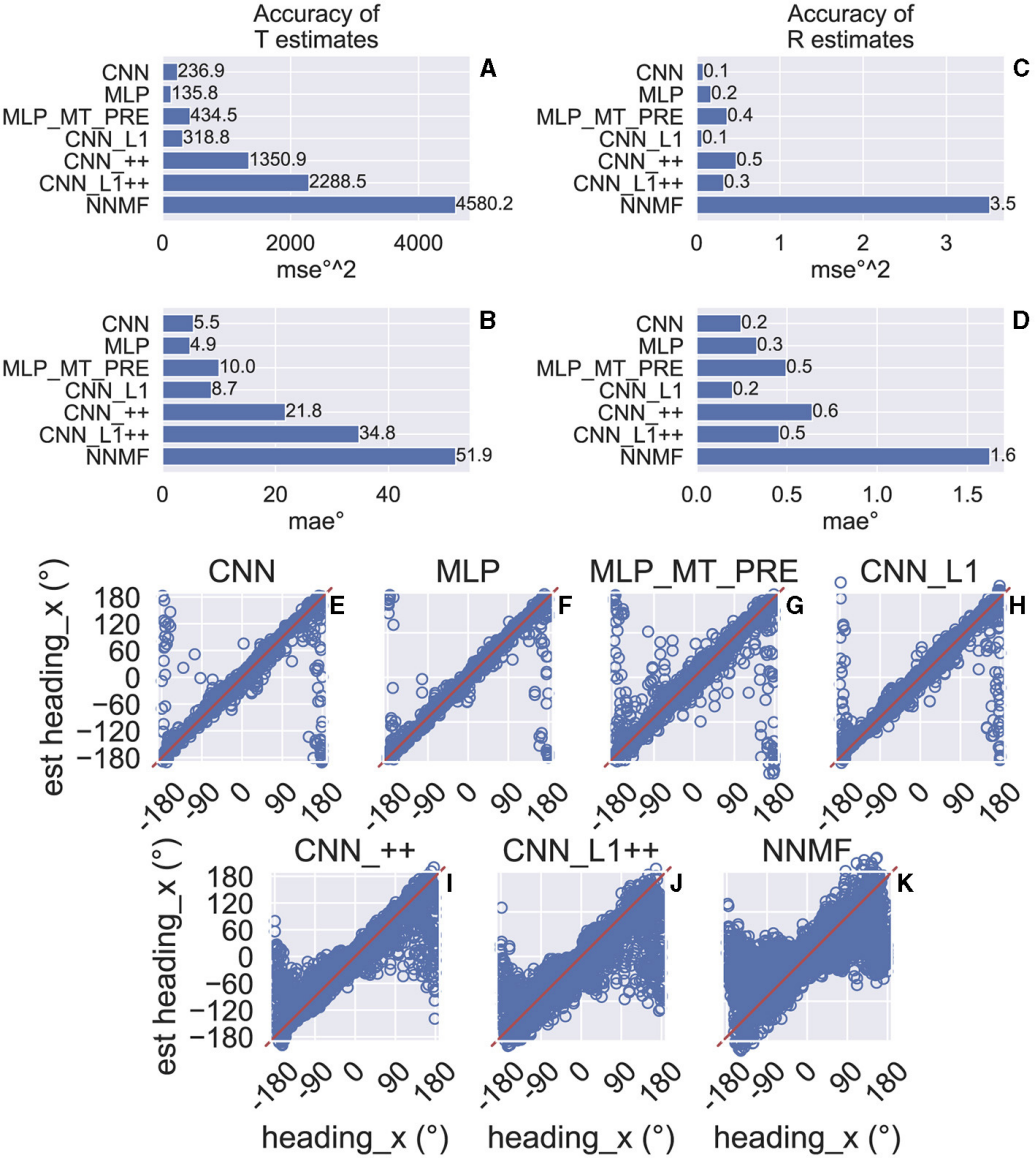
Test accuracy on the TR360 optic flow dataset obtained by the CNN and NNMF models. MSE represents mean squared error and MAE represents mean absolute error. **(A, B)** Accuracy of translational self-motion estimates achieved by each model. **(C, D)** Accuracy of rotational self-motion estimates achieved by each model. **(E–K)** Scatter plots focus on the model predictions for individual optic flow test samples in the case of azimuthal translation (“heading_x”). The x axis corresponds to the true value, the y axis corresponds to the value predicted by the model, and the red curve shows the unity line (no error). Note that headings of 180° and –180° correspond with the same direction (backward).

We assessed the accuracy with which the neural networks estimate the 3D translation and rotation of the simulated observer on a test set comprising 3,015 novel optic flow stimuli that the models did not encounter during training. The CNN estimates translation and rotation with a high degree of accuracy—the CNN achieves mean absolute errors (MAEs) of 5.5° (Figure 5B) and 0.2° (Figure 5D), respectively. The mean squared error (MSE) in estimating the corresponding self-motion parameters are 236.9°^2^ (Figure 5A) and 0.1°^2^ (Figure 5C). As Figures 5A–D indicates, the accuracy achieved by the MLP is comparable to that of the CNN. The MLP that integrates the MT-like motion representation rather than optic flow (MT_MT_PRE) garners approximately double the MSE and MAE. While CNN_L1 performs similarly to both the CNN and MLP, networks with the non-negative weights estimate translation and rotation with far less accuracy (CNN_++, CNN_L1++; Figures 5A–D). Inspection of the individual predictions reveals qualitative differences: whereas CNN and MLP yield a high concentration of estimates near true values along the unity line (Figures 5E, F), predictions from the CNN_++, CNN_L1++ are more dispersed and deteriorate markedly when estimating backward translation (≈ ± 180°). Combining L1 regularization and the non-negative weight constraint does not improve accuracy compared to CNN_++ (Figure 5J).

For comparison, we simulated the original NNMF model of Beyeler and colleagues (Beyeler et al., 2016). To do this, we fit a linear regression to the NNMF model MSTd activations obtained on the training set stimuli, and, similar to the CNNs, we assessed the accuracy of estimates obtained on the test set. Figures 5A–D show that the NNMF model yields substantial translation and rotation errors. For example, in the case of translation NNMF garners approximately twice the MSE and MAE obtained by the CNNs that yield the least accurate estimates (CNN_++, CNN_L1++). Figure 5K reveals that the individual NNMF predictions more closely resemble those produced by CNN_++ and CNN_L1++ than those without the non-negativity constraint.

The poor accuracy of self-motion estimates decoded from NNMF is somewhat surprising given that Beyeler et al. (2016) report only ≈ 5° and 1° mean error when decoding translation and rotation, respectively, from the optic flow stimuli of Ben Hamed et al. (2003). These are novel stimuli not used to fit the model. The discrepancy in accuracy could stem from the fact that the Ben Hamed stimuli were considerably simpler than the TR360 dataset simulated here. The Ben Hamed stimuli consist of two separate datasets: either pure translation within ±45° of straight-ahead (BenHamedT dataset) or pure rotation (BenHamedR dataset). This contrasts with the TR360 dataset that contains combined translation and rotation along any 3D direction. Without refitting the models (i.e., the weights reflect learning on the more general TR360 dataset), we decoded self-motion from the CNNs and the NNMF model on the Ben Hamed datasets to determine whether task difficulty could account for the large difference in accuracy. Following Beyeler et al. (2016), we decoded from the NNMF model by fitting a separate linear regression for each self-motion label and assessing the mean validation accuracy through a 10-fold cross validation procedure. We carried out this process for the BenHamedT and BenHamedR datasets independently. To facilitate comparison with the CNNs that were trained end-to-end on the TR360 dataset with a supervised learning paradigm, we lesioned the weights between the last hidden layer and the output layer and fit separate linear regressions to the last hidden layer activations through the same 10-fold cross validation procedure that was used with the NNMF model. Consistent with Ben Hamed et al. (2003); Beyeler et al. (2016), we decoded from a random sample of 144 neurons in each model unless the last hidden layer of a network did not have this many responsive neurons. In this case, we decoded from all responsive neurons.

**Figure 6:**
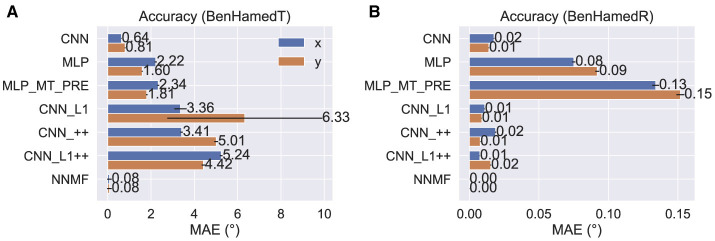
Accuracy of each model on the BenHamedT **(A)** and BenHamedR **(B)** datasets. The BenHamedT dataset consists of optic flow corresponding to translation-only self-motion and the direction of translation varies –45−45° in both x and y. The BenHamedR dataset consists of optic flow corresponding to rotation-only self-motion with –10−10°/s speeds. Error bars show 95% confidence intervals (CIs) on the MAE estimated by 10-fold cross-validation.

Figure 6 shows the MAE obtained by cross-validation when the models estimate the horizontal (x) and vertical (y) translational (heading) components in the BenHamedT dataset (Figure 6A) and the corresponding rotational components in the BenHamedR dataset (Figure 6B). Both NNMF and the neural networks estimated self-motion with a high degree of accuracy—MAEs of several degrees or less. The substantial gap in accuracy between the CNN variants with and without the non-negativity constraint (CNN_++ and CNN_L1++) obtained on the TR360 dataset (Figure 5) largely vanishes on the Ben Hamed datasets. It is important to consider that the BenHamedT dataset contains headings ranged from –45° to 45° in both the horizontal and vertical directions and BenHamedR dataset contains rotational speeds that range between –10 and 10°/s. Together, these findings suggest that the NNMF model encodes optic flow accurately when decoding pure translation and rotation over limited extents, but incurs substantial error when estimating self-motion from more complex combinations of translation and rotation. The fact that most models yield highly accurate estimates on Ben Hamed datasets supports the notion that task difficulty explains the discrepancy between NNMF accuracy on the TR360 and Ben Hamed datasets.

To establish whether the disparity in accuracy achieved by NNMF across the TR360 and Ben Hamed dataset stems from its non-negative and sparse “parts-based” representation, we applied the PCA algorithm to the MT optic flow activations and performed the same decoding procedure. PCA is another dimensionality reduction algorithm that does not enforce non-negativity and does not yield a parts-based encoding. We found that PCA configured with 64 basis vectors (i.e., same as NNMF) yields the same pattern of results as NNMF—4,962.1°^2^ and 5.34°^2^ MSE when estimating translation and rotation, respectively, on TR360 test samples as well as 0.16° and ≈0° MAE when estimating translation and rotation, respectively, on the Ben Hamed stimuli. We conclude that the poor NNMF generalization on the TR360 dataset is not necessarily the consequence of a non-negative and sparse encoding.

### Translation tuning profiles

**Figure 7:**
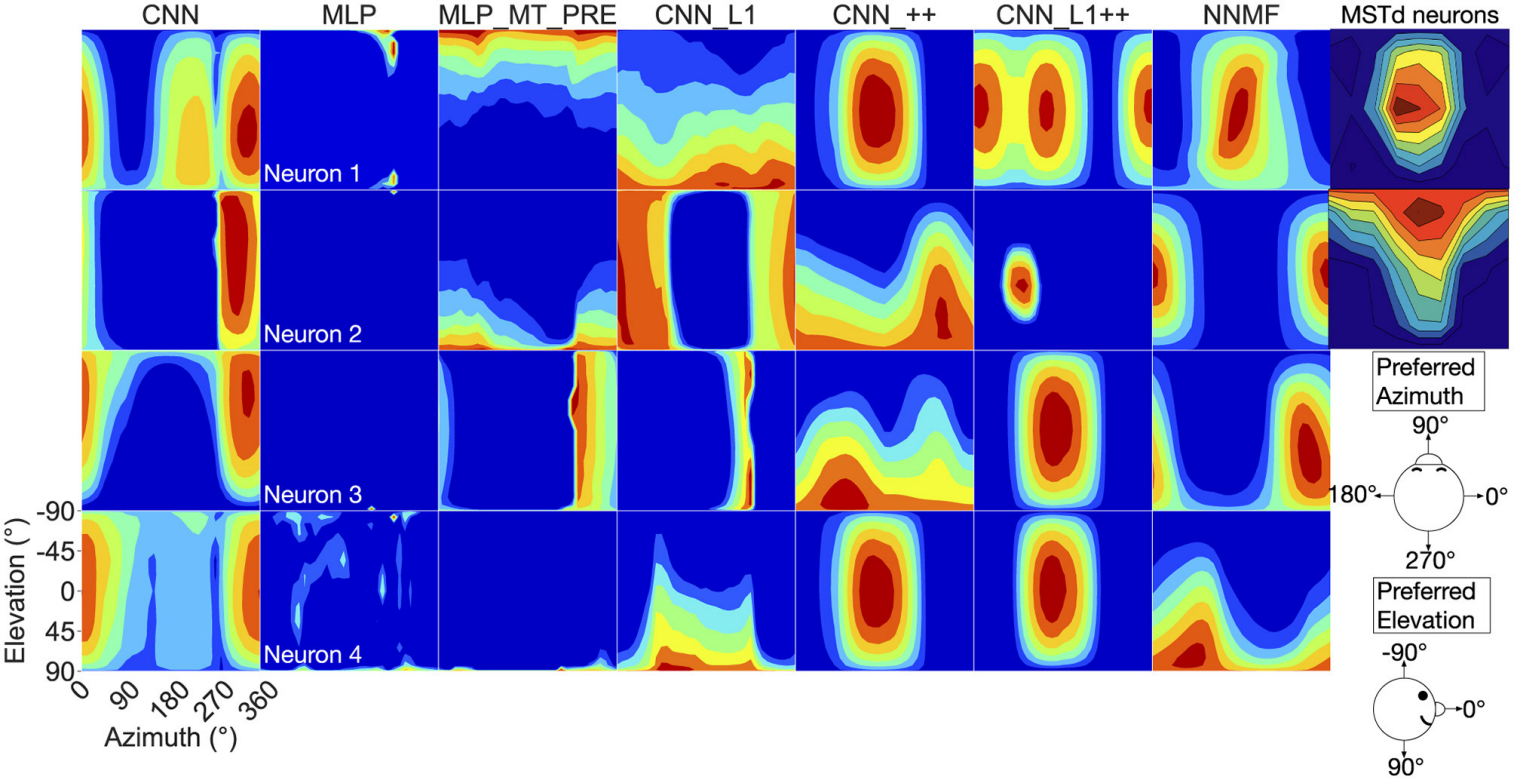
3D translation tuning profiles for four randomly selected neurons (rows) in each model (columns). Translation directions are expressed respect to azimuth and elevation angle (°). Warmer colors correspond to stronger activation to specific combinations of azimuth and elevation angles and cooler colors correspond to weaker activation. The translation tuning profiles for two MSTd neurons from Takahashi et al. (2007) are depicted in the rightmost column. Copyright 2007 Society for Neuroscience. The bottom-right diagram schematizes the translation direction coordinate system.

Now we turn our analysis to characterizing the tuning of model units to translational optic flow and drawing comparisons with primate MSTd. The heatmaps in Figure 7 show the translation tuning profile of four units randomly selected from each model alongside the tuning profiles of two MSTd neurons from Takahashi et al. (2007). We generated each heatmap by considering the neural responses of a single model unit to 512 translational optic flow stimuli with regularly spaced azimuths and elevations (11.25° steps) from the TestProtocolT diagnostic dataset (see Materials and Methods). To facilitate comparison with existing neurophysiological and modeling work, we adopt the same coordinate system as Takahashi et al. (2007) and Beyeler et al. (2016) where 0° and 180° azimuth correspond to leftward and rightward translation, respectively, and –90° and 90° elevation correspond to upward and downward translation, respectively (see bottom-right schematic in Figure 7). In some cases the tuning profiles of single model units share qualitative similarities with the MSTd neuron exemplars (e.g., MSTd Neuron 2, NNMF Neuron 4, CNN_++ Neuron 2).

**Figure 8:**
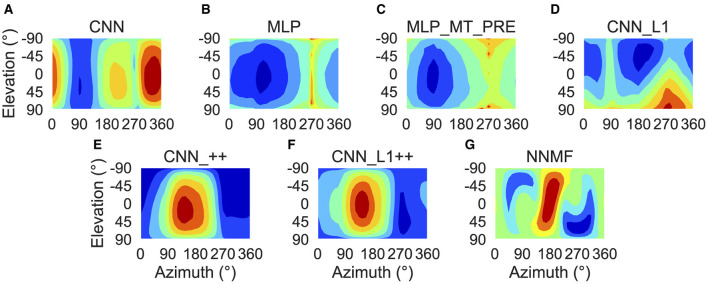
Population translation tuning profiles for each model. **(A–G)** Same plotting conventions and coordinate system as Figure 7.

To characterize translation responses more generally across the population, we summed the activations obtained to each translation direction and generated population translation tuning profiles for each model (Figure 8). The CNN population (Figure 8A) produces the highest activation to ≈300° azimuths (backward-right translation). The MLP, MLP_MT_PRE, and CNN_L1 models (Figures 8B–D) respond maximally to 270° azimuths (backward translation) and minimally to 90° azimuths (forward translation). The activation in both MLP models is tightly concentrated at upward and downward elevations and weak around 0° elevations (Figures 8B, C), whereas in the CNN_L1 model there is an asymmetric bias toward downward translation (Figure 8D). The CNNs with the non-negativity weight constraint yield peak responses (Figures 8E, F) around 135° azimuth (forward-left translation), which are close to the peak produced by the NNMF model (Figure 8G).

### Translation direction preferences

**Figure 9:**
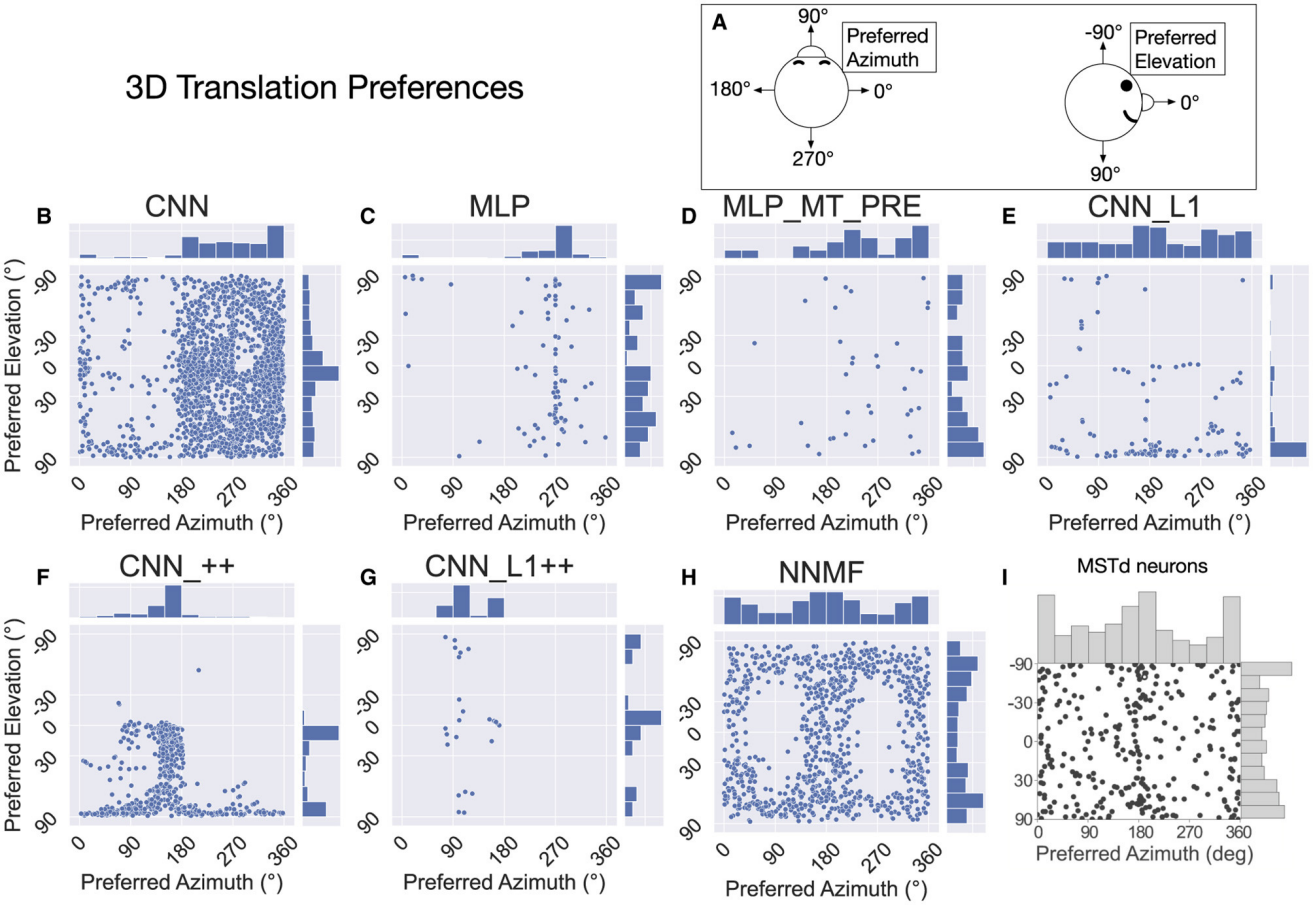
The 3D translation direction preferences of single neurons in each model. **(A)** Schematic depicting the coordinate system, which is the same as in Figure 7. **(B–H)** The preferred direction of each neuron is expressed as its preferred combination of azimuth and elevation angles. The bar charts on top and to the right of each scatter plot depict histograms (12 bins, bin width: 30°), showing the marginal distribution of neurons with different preferred azimuth and elevation angles, respectively. **(I)** 3D translation direction preferences of MSTd neurons from Takahashi et al. (2007). Copyright 2007 Society for Neuroscience.

Following Takahashi et al. (2007) and Beyeler et al. (2016), we characterized the translation tuning preference of each model unit using population vector decoding (see Materials and Methods). Each scatter plot in Figure 9 shows the preferred translational azimuth and elevation angle of single neurons in a particular model. The histograms to the right and above each scatter plot show the marginal distribution of preferred azimuth and elevation angles, respectively, across the population. From the scatter plots and histograms it is clear that the translation preferences of NNMF model units (Figure 9H) exhibit the greatest consistency with those of MSTd neurons (Figure 9I). A key characteristic of MSTd preferences for translational azimuth angle is the relative paucity of neurons that prefer fore-aft self-motion directions (90°, 270°) compared to lateral directions (0°, 180°; Figure 9I). While few units in the CNN and MLP models prefer ≈90° azimuth (forward) translation, many units in these models possess ≈270° (backward) azimuthal preferences, a departure from the MSTd data. The exceptions are the models with the non-negativity constraint (CNN_++, CNN_L1++), in which most neurons prefer forward or leftward translation, respectively. The preference for backward translation in the CNN, MLP, MLP_MT_PRE, CNN_L1 models is consistent with their population tuning profiles (Figures 8A–D). None of the models other than NNMF capture the symmetric preference for large elevation angles.

Interestingly, even though the distributions of preferred translation azimuth and elevation in the CNN model mismatch the MSTd data (compare Figures 9B and I), the percentage of neurons that have preferences within 30° of the lateral, fore-aft, and vertical axes of translation exhibit a high degree of consistency with the neural data of Takahashi et al. (2007) (Table 4). The neurons in the CNN_++ and NNMF models demonstrated a similar qualitative pattern whereby there is a relative paucity of neurons within 30° of the fore-aft axis of translation.

**Table 4:** The percentage of neurons in each model with preferred translation directions within 30° of the lateral (leftward-rightward), fore-aft (forward-backward), and vertical (upward-downward) axes.

**Study/Model**	**Lateral (0°, 180°)**	**Fore-Aft (90°, 270°)**	**Vertical (-90°, 90°)**
Takahashi et al. (2007)	**57/307 (19%)**	**20/307 (7%)**	**76/307 (25%)**
CNN	**300/2,383 (13%)**	**195/2,383 (8%)**	**591/2,383 (25%)**
MLP	1/86 (1%)	19/86 (22%)	33/86 (38%)
MLP_MT_PRE	1/39 (3%)	2/39 (5%)	19/39 (49%)
CNN_L1	4/137 (3%)	3/137 (2%)	100/137 (73%)
CNN_++	459/1,094 (42%)	35/1,094 (3%)	390/1,094 (36%)
CNN_L1++	8/27 (30%)	8/27 (30%)	11/27 (41%)
NNMF	64/896 (7%)	4/896 (0.4%)	392/896 (44%)

Each bold entry shows the closest percentage to that reported by Takahashi et al. (2007) for MSTd.

In summary, the NNMF model best approximates MSTd translation preferences. While the translation tuning profiles of the CNNs with the non-negativity constraint most closely resemble those of NNMF (Figures 7, 8), these CNNs do not reproduce the pattern of 3D translation preferences across the population (Figure 9). The other neural network models deviate from MSTd preferences due to the predominance of units that prefer backward translation.

### Translation tuning width

**Figure 10:**
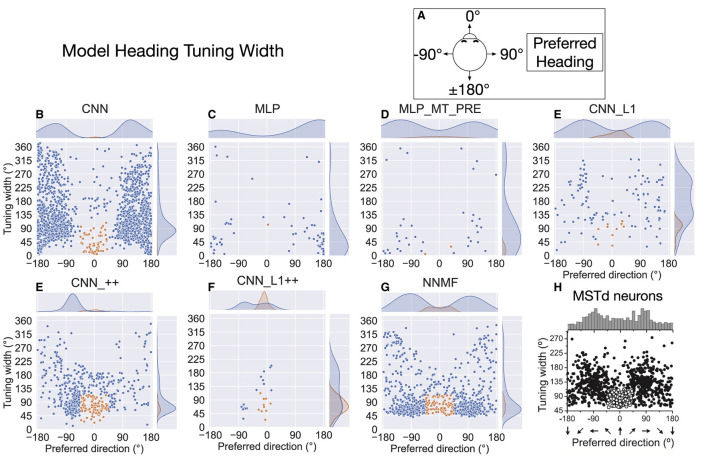
Translation tuning width at half maximum of each model neuron. **(A)** Schematic of coordinate system, which is different than in Figure 9 to facilitate comparison between model tuning widths **(B–G)** and MSTd data from Gu et al. (2010) **(H)**. Filled curves on the top and right side represent the marginal kernel density estimates. As depicted in **(H)**, scatter plots show neurons with preferred heading within 45° of straight-ahead and tuning widths < 115° in orange. Panel **(H)** reprinted from Gu et al. (2010) with permission from Elsevier.

The preceding analysis characterizes the translation direction that elicits the maximal response in each unit, but it does not capture the spatial extent of the tuning. Figure 10 plots the tuning width at half maximum for single units in each model (see Materials and Methods). As indicated in Figure 10A, we adopt a different coordinate system to facilitate comparison with MSTd translation tuning width data from Gu et al. (2010) (Figure 10H). In this coordinate system, the –90° and 90° correspond to leftward and rightward translation, respectively, and 0° and ±180° correspond to forward and backward translation, respectively. The kernel density estimates on the top of each scatter plot show bimodal distributions in all the models except for the CNNs with the non-negativity constraint (CNN_++, CNN_L1++). The peaks in NNMF distribution (Figure 10G) peaks appear at ±90°, which most closely matches in the MSTd data (Figure 10H). The kernel density estimates reflect the aforementioned backward bias in CNN, MLP, MLP_MT_PRE, and CNN_L1 translation preferences (±180°). Neurons in the CNN model demonstrate the most variability in their tuning widths, particularly among those tuned to backward translation. NNMF, CNN, and CNN_L1, but not the MLPs, contained a cluster of neurons tuned within 45° of the straight-ahead that showed narrower tuning widths (orange markers). However, only the NNMF model exhibits the tendency of MSTd neurons for the minimum tuning widths to be at least 45°, regardless of the preferred direction.

### Heading discriminability and strength of tuning

**Figure 11:**
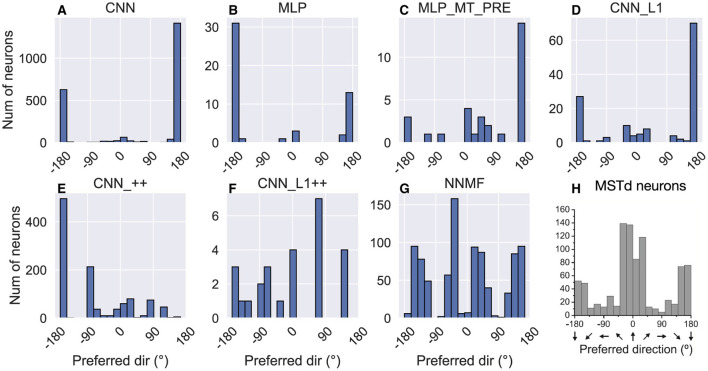
Histograms (18 bins, width: 20°) showing peak heading discriminability of units in each model **(A–G)**. Optic flow stimuli involve translation in the horizontal plane without variations in elevation or rotation. Coordinate system of Figure 10 is used. **(H)** Peak heading discriminability of MSTd neurons from Gu et al. (2010). Panel **(H)** reprinted from Gu et al. (2010) with permission from Elsevier.

While MSTd neurons across the population exhibit lateral (leftward and rightward) bias in their translation preference, they possess peak discriminability for straight-ahead, and to a lesser extent, backwards headings (Gu et al., 2010) (Figure 11H). To assess the extent to which the models reproduce this tendency, we computed the heading at which each model units demonstrates maximal discriminability (see Material and Methods). The CNN, MLPs, and CNN_L1 models yield qualitatively similar distributions, with peak discriminability at backward headings (≈ ± 180°). While a large subpopulation of MSTd neurons does exhibit peak heading discriminability to backward headings, most neurons exhibit peak heading discriminability to forward headings (Figure 11H). While the NNMF model yields a peak close to straight-ahead and lesser peaks at greater eccentricities (Figure 11G), its four distinct peaks are punctuated by sharp drops in peak discriminability at the cardinal axes (0°, 90°, ±180°), a pattern that does not appear in the MSTd data (Figure 11H).

**Figure 12:**
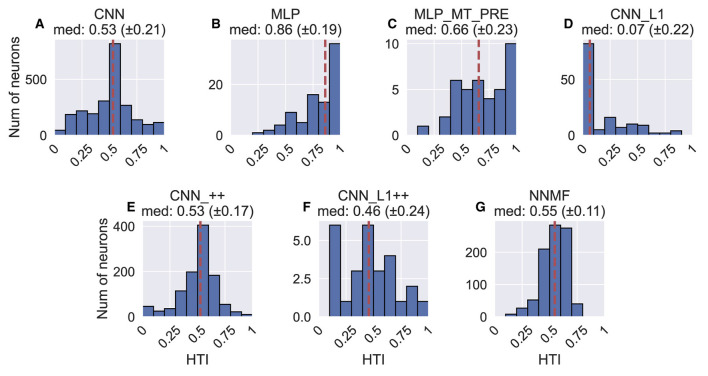
Histograms (10 bins) showing the heading tuning index (HTI) of units in each model. **(A–G)** The values above each plot show the median HTI across the population as well as its standard deviation. The dashed vertical line corresponds to the median HTI.

Figure 12 shows the heading tuning index (HTI) computed on the TestProtocolT diagnostic dataset (see Materials and Methods). The HTI quantifies the strength of a unit's heading tuning and ranges between 0 and 1 (Gu et al., 2006). A value of 0 indicates weak heading tuning while a value of 1 indicates strong heading tuning (i.e. unit selectively activates to only a single heading). Gu et al. (2006) obtained a mean HTI of 0.48 ± 0.16 SD for their MSTd population. Due to the differences in the HTI distribution shapes, we report median HTIs for each model population. The CNN (median ± SD, 0.53 ± 0.21), CNN_++ (0.53 ± 0.17) and NNMF (0.55 ± 0.11) demonstrate the best agreement with the MSTd HTIs. The variability in the CNN_++ (0.17) is closest to the MSTd population (0.16). Consistent with their narrow tuning widths (Figures 10C, D) and concentrated peak activations across the population (Figures 8B, C), MLP and MLP_MT_PRE neurons are more heading selective (respective 95% CIs: [0.82, 0.90]; [0.58, 0.73] ) than the CNN [0.52, 0.54] and CNN_L1 [0.03, 0.10]. Neural populations in CNN_L1 [0.03, 0.10] exhibit notably weaker overall heading selectivity.

In summary, the correspondence between the models and MSTd heading discriminability and strength of tuning is mixed. While the CNN, CNN_++, and NNMF models capture key aspects of MSTd heading selectivity (Figure 12), there is poor correspondence with with respect to peak heading discriminability (Figure 11).

### Rotation direction preferences

**Figure 13:**
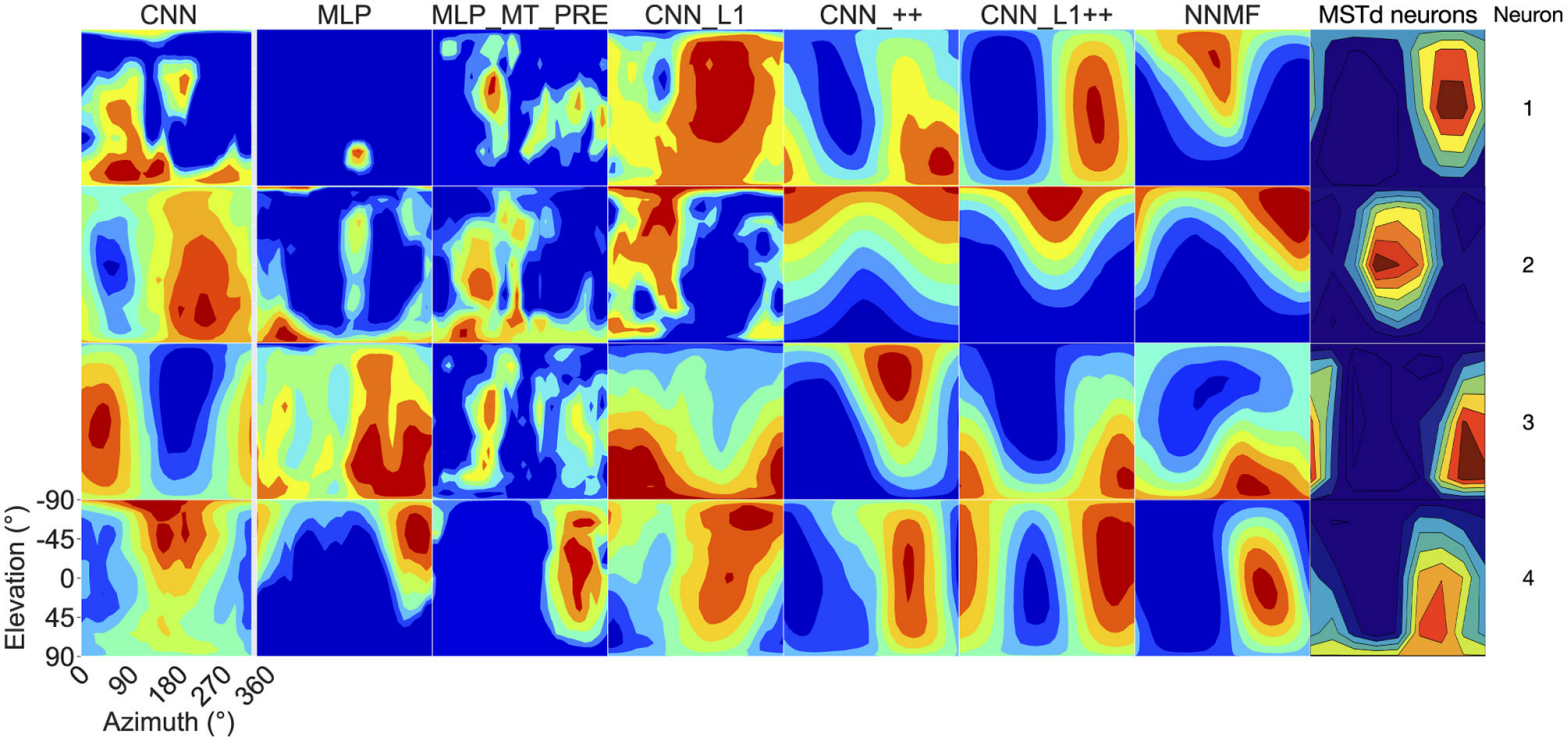
3D rotation tuning profiles for four randomly selected neurons (rows) in each model (columns). Same format and coordinate system as Figure 7. The rotation tuning profiles for four MSTd neurons from Takahashi et al. (2007) are depicted in the rightmost column. Copyright 2007 Society for Neuroscience.

**Figure 14:**
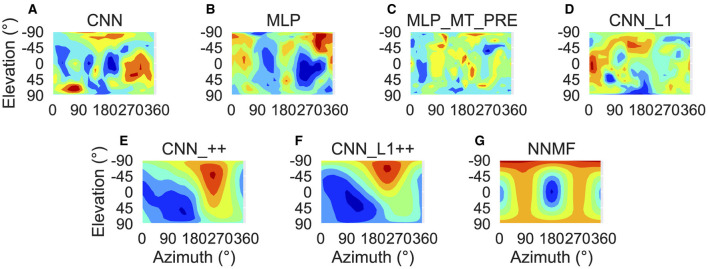
Population rotation tuning profiles for each model. **(A–G)** Same plotting conventions and coordinate system as Figure 8.

Next we focus on model tuning to rotational optic flow. Since we compare model properties to the MSTd data of Takahashi et al. (2007), we once again adopt their coordinate system (see Figure 13 lower left; Figure 9). Analogous to the procedure used for translation, we generated each heatmap by considering the neural responses of a single model unit to 514 rotational optic flow stimuli with regularly spaced azimuths and elevations (11.25° steps) from the TestProtocolR diagnostic dataset (see Materials and Methods). Each pair of preferred azimuth and elevation angles now indicate the direction about which visual rotation induces the maximal activation. Figure 13 shows a sample of the diverse rotation tuning profiles derived from the single unit activations to the TestProtocolR diagnostic dataset alongside MSTd exemplars (Figure 13, right column). It is noteworthy that NNMF and the CNNs with the non-negativity constraint tend to profile unimodal rotational tuning profiles, which is a key characteristic of the MSTd neurons from Takahashi et al. (2007). By contrast, the CNN, MLPs, and CNN_L1 model yield multimodal profiles in a number of instances, which do not resemble the MSTd neurons. These characteristics are also apparent at the population level tuning profiles, shown in Figure 14. As is the case with translation (Figure 8), the population rotation profiles of the CNN_++ and CNN_L1++ neural network models bear the closest similarity to NNMF — in this case, this involves increased population activation to rotation about the higher elevation angles.

**Figure 15:**
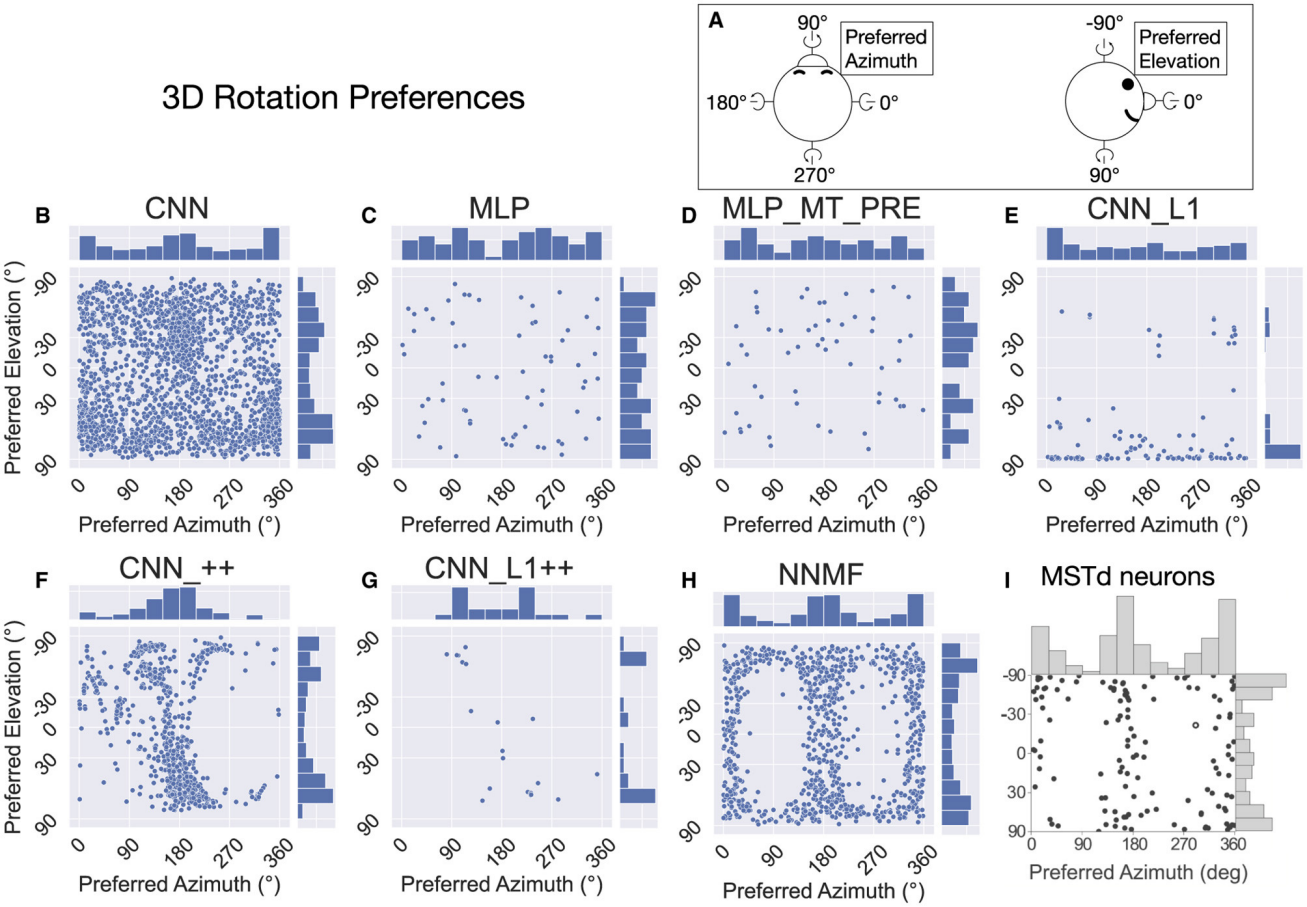
The 3D rotation direction preferences of single neurons in each model. Same format as Figure 9. **(A)** Schematic depicting the the coordinate system. **(B–H)** The rotation azimuth and elevation angle preference of each model neuron. **(I)** 3D rotation direction preferences of MSTd neurons from Takahashi et al. (2007). Copyright 2007 Society for Neuroscience.

Figure 15 presents the preferred azimuth and elevation about which neurons in each model prefer rotational optic flow. We determined the rotation preference of each neuron using population vector decoding from the activations garnered on the TestProtocolR diagnostic dataset. Consistent with Beyeler et al. (2016) and MSTd rotation preferences (Figure 15I), the most neurons in the NNMF model population peak at around 0/360° (left) and 180° (right) in azimuth preferences. The marginal histograms for azimuth and elevation garnered by the CNN model resemble those from the neural data (compare Figures 15B and I). Unlike the MSTd population, however, CNN rotation preferences are broadly dispersed in azimuth and elevation, whereas both the MSTd population and NNMF possess few neurons tuned to 90° and 270° azimuths. The CNN_++ model possesses the MSTd-like bimodal distribution of elevation angle preferences and preponderance of neurons tuned to 180° azimuths, yet it lacks many neurons tuned to 0/360° azimuths. The other models do not demonstrate consistency with the rotation tuning preferences in the MSTd population. Overall, rotation preferences in the NNMF and CNN models produce the best agreement with those of MSTd neurons.

Table 5 summarizes the number of neurons that exhibit rotation tuning within 30° of the cardinal yaw, pitch, and roll axes. Approximately one quarter of neurons in the neurophysiological study of Takahashi et al. (2007) exhibited rotation preferences close to the yaw and pitch axes and almost none exhibited a preference close to the roll axis. While a number of models possessed a similar percentage along one axis, none captured the tuning pattern across all axes of rotation.

**Table 5:** The percentage of neurons in each model with preferred rotation directions within 30° of the vertical (yaw), lateral (pitch), and depth (roll) axes.

**Study**	**Yaw**	**Pitch**	**Roll**
Takahashi et al. (2007)	36/127 (28%)	27/127 (21%)	**1/127 (1%)**
CNN	736/2,337 (31%)	214/2,337 (9%)	109/2,337 (5%)
MLP	17/66 (26%)	1/66 (2%)	8/66 (12%)
MLP_MT_PRE	**17/56** (30%)	4/56 (7%)	4/56 (7%)
CNN_L1	92/134 (69%)	2/134 (1%)	**0/134 (0%)**
CNN_++	404/991 (41%)	**93/991 (9%)**	41/991 (41%)
CNN_L1++	15/22 (68%)	2/22 (9%)	**0/22 (0%)**
NNMF	448/896 (50%)	75/896 (8%)	4/896 (0.4%)

Each bold entry shows the closest percentage to that reported by Takahashi et al. (2007) for MSTd.

### Difference between translation and rotation preference

**Figure 16:**
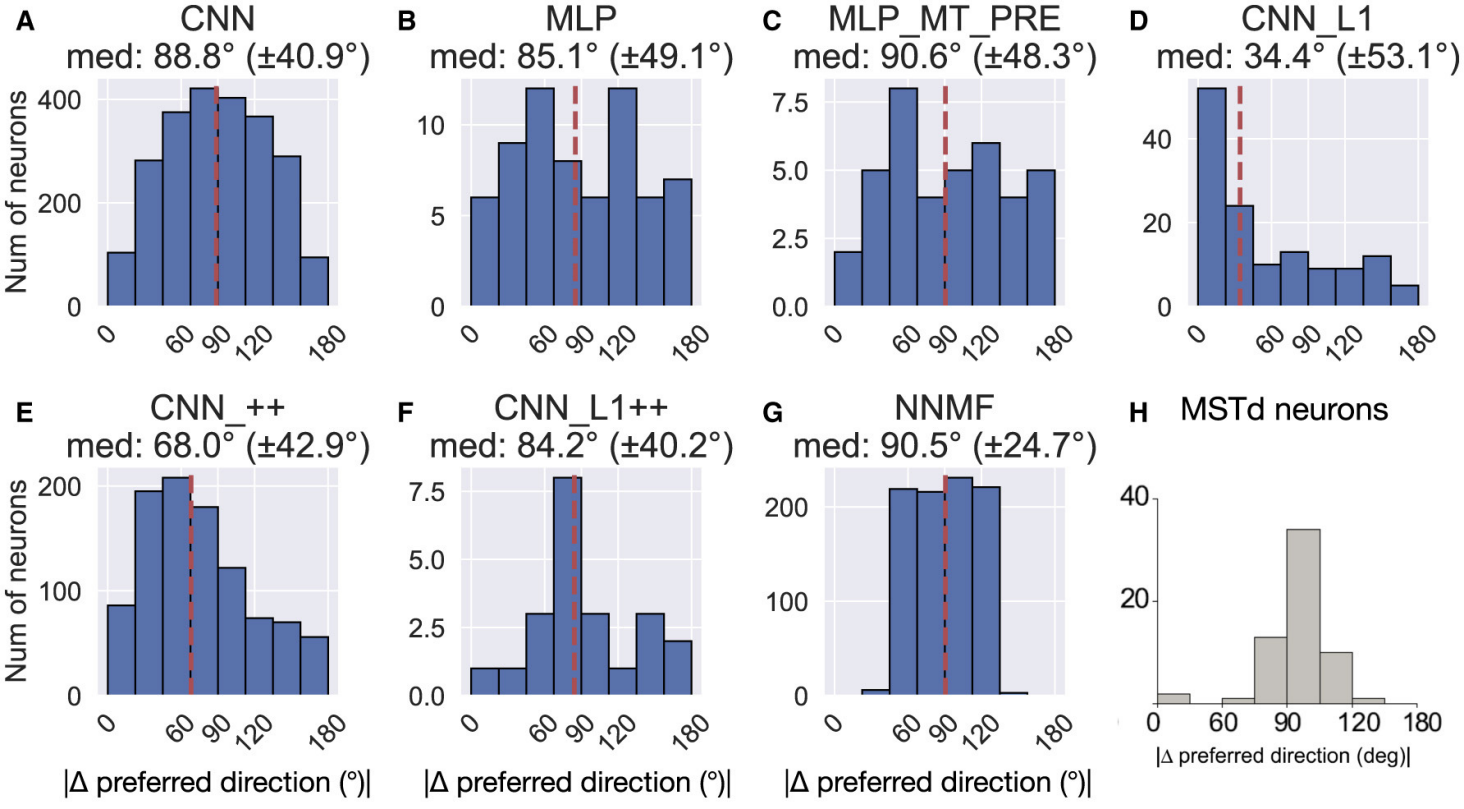
Difference between each neuron's translation and rotation preference. **(A–G)** The values above each plot show the median difference across the population as well as its standard deviation. **(H)** Difference in translation and rotation preference in MSTd population from Takahashi et al. (2007). Copyright 2007 Society for Neuroscience. The dashed vertical line corresponds to the median difference in preference.

Figure 16 shows the difference in each model unit's preferred translation and rotation angle. With the exception of CNN_L1, all the models yield median differences of ≈90°, consistent with MSTd neurons from Takahashi et al. (2007) (Figure 16H). However, there is considerable variability among the neurons within each model. Similar to MSTd, NNMF neurons exhibit a more distinct, albeit wider, peak at ≈90° (Figure 16G). CNN_L1++ does as well, though the overall number of neurons that contribute is quite small.

### Rotation tuning index

**Figure 17:**
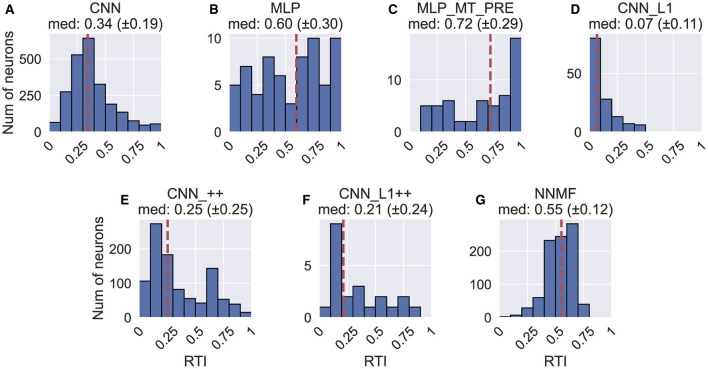
Histograms (10 bins) showing the rotation tuning index (RTI) of units in each model. **(A–G)** Same format as Figure 12.

Figure 17 shows the rotation tuning index (RTI) of each model unit. The RTI measures the strength of a neuron's rotation tuning on a scale between 0 and 1. We compute RTI in the same way as HTI (Equation 10), except activations correspond to the TestProtocolR diagnostic dataset that contains rotation-only optic flow samples and the labels in Equation 10 now correspond to rotation. Figure 17 reveals that the rotation tuning is weakest in the CNN_L1 model (median 95% CI: [0.05, 0.09]). The CNN [0.34, 0.35], CNN_++ [0.24, 0.27], CNN_L1++ [0.12, 0.31] models exhibit more moderate rotation tuning. NNMF neurons exhibited comparatively stronger rotation tuning [0.54, 0.56]. Interestingly, while the MLPs produce broad RTI distributions, both models contain a subpopulation with exquisitely selective neurons (RTI≈1).

In summary, the CNN marginal distributions of rotation azimuth and elevation preferences (Figure 15B) demonstrate consistency with the MSTd data (Figure 15I), though the overall 3D distribution bears less similarity. The CNN diverges from the neural data in the prevalence of multimodal tuning profiles (Figures 13, 14) and the high variability in the differences between translation and rotation preferences. The CNN with the non-negativity constraint (CNN_++) produces MSTd-like unimodal tuning profiles and reproduces key characteristics of the 3D distribution MSTd rotation preferences, but yields a broad distribution of differences between translation and rotation preferences. The NNMF model reproduces most of the key rotation tuning characteristics of MSTd neurons out of the models simulated.

### Sparseness

**Figure 18:**
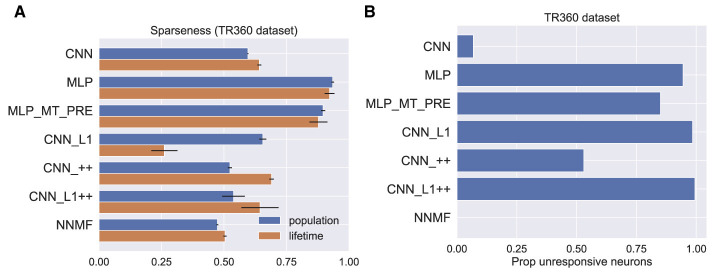
Measures of sparseness in the computational models computed on the TR360 test set. **(A)** Population and lifetime sparseness metrics. Error bars show 95% confidence intervals on the mean sparseness across neurons (population) or stimuli (lifetime) in each model. **(B)** The proportion of neurons that did not elicit responses to any of the stimuli in the TR360 test set.

Beyeler et al. (2019) propose that sparseness may represent a defining property of optic flow encoding in area MSTd. Following Beyeler et al. (2016), we computed sparseness metrics based on the model activations to optic flow samples in the TR360 test set (Vinje and Gallant, 2000). The population sparseness measures the proportion of neurons that activate to a single stimulus. The lifetime sparseness measures the proportion of stimuli in the dataset to which a single neuron activates. Both metrics range from 0 to 1. Zero indicates a dense code where a stimulus activates every neuron. One indicates a localist code where a stimulus activates only a single neuron—a grandmother cell representation. Hence, an intermediate value indicates a sparse code wherein a subset of neurons activate to a stimulus. Figure 18A shows that the CNNs and NNMF models possess comparably moderate sparseness in activations to optic flow in the TR360 test set according to both metrics. It is notable that both MLPs possesses a considerable degree of sparseness, which indicates a more localist code.

We examined another phenomenon that promotes sparseness in deep neural networks that use the common ReLU activation function as we do here. Because neurons that implement the ReLU activation function output zero activation when their input is subthreshold, it is possible throughout the course of training for a neuron's afferent weights to be set so that even the largest possible inputs cannot activate the neuron. This is known as the “dying ReLU” (Lu et al., 2019). While this is often referred to as a “problem” in the machine learning literature, this phenomenon may confer benefits, such as forcing the neural network to encode stimuli in a subset of available neurons. This may encourage sparseness, simplicity, and efficiency in the stimulus encoding (Glorot et al., 2011). Figure 18B shows the proportion of unresponsive neurons in each model, which we define as the proportion of neurons that never activate to any optic flow stimulus in the TR360 test set. It is striking that only 7% of the neurons in the CNN model were unresponsive to the TR360 test set stimuli, whereas in other neural network models, more than 50% of the neurons were unresponsive. A large percentage of neurons were unresponsive in the MLP (94% ) and MLP_MT_PRE (85%) models, which is consistent with their sparseness metrics (Figure 18A). The CNN_L1 and CNN_L1++ models both possess larger percentages of unresponsive neurons, 98% and 99%, respectively, than CNN_++ (53%), which suggests that the L1 regularization values used in the accuracy-optimized models promote sparseness in the optic flow encoding. Interestingly, all the neurons in the NNMF responded to at least one stimulus—it contains no unresponsive neurons. This likely stems from the computational goal of NNMF: to learn a small set of basis vectors that may be used to reconstruct the stimulus dataset.

### Motion tuning in early convolutional layer

**Figure 19:**
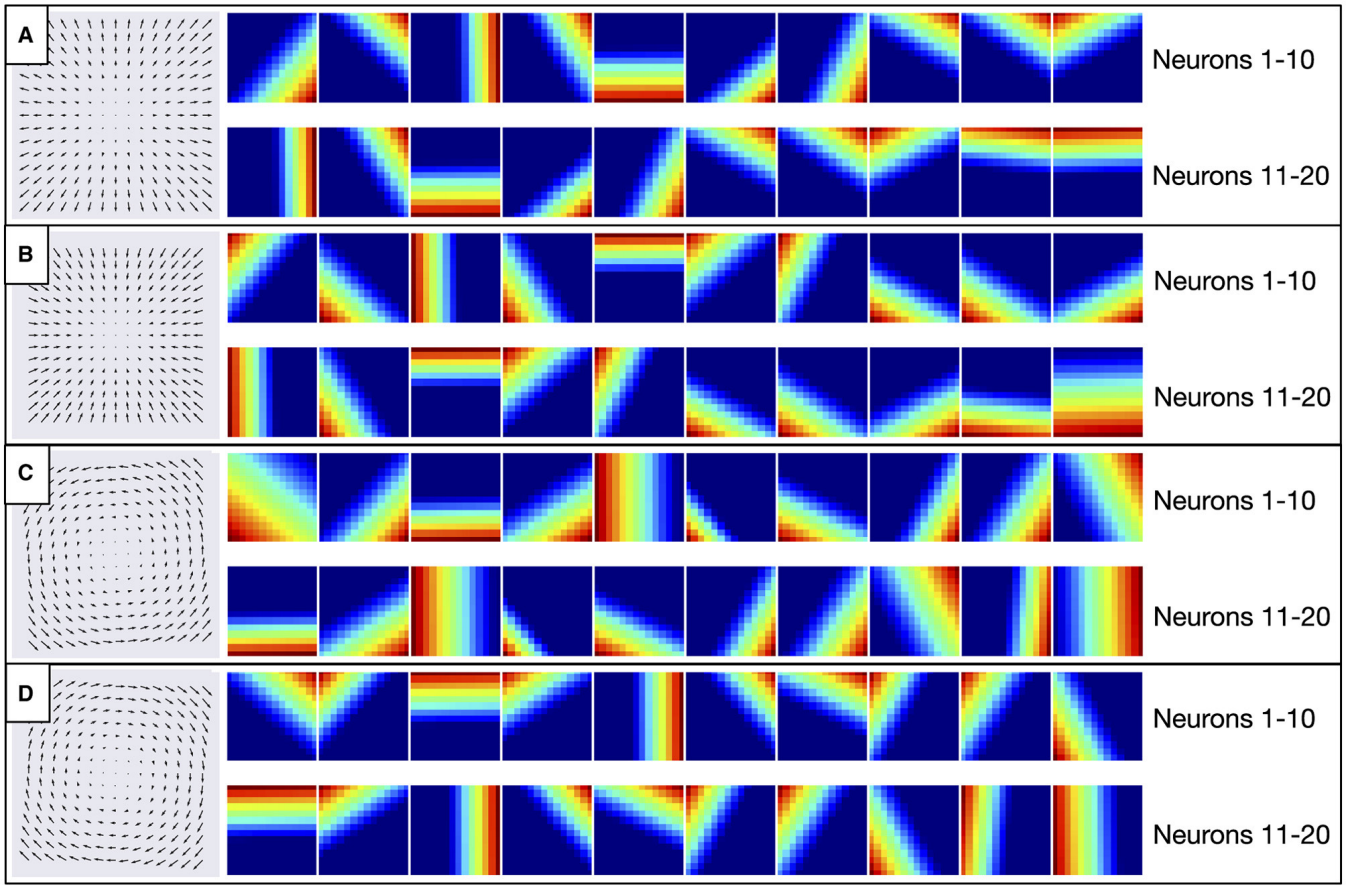
Activation of 20 exemplar filters from the first layer of CNN model to the optic flow patterns depicted in the left column. **(A–D)** Within each panel, individual heatmaps show spatial activations of single filters to the corresponding optic flow pattern. Each activation map is normalized and warmer colors indicate higher activation within corresponding regions of the optic flow field. The same 20 filters are shown between panels (e.g., top-left heatmap in each panel corresponds to Neuron 1).

The search to optimize each CNN model selected a single convolutional layer with relatively small 2 × 2 filter sizes (Table 3). This filter size corresponds to ≈12° RF sizes, which is common in area MT (Tanaka et al., 1986). Given that optic flow tuned MSTd neurons receive substantial input from area MT (Maunsell and Van Essen, 1983), this raises the intriguing possibility that neurons in the early convolutional layer of the CNNs may develop MT-like uniform direction tuning within the RF (Born and Bradley, 2005). Another possibility is that early neurons could learn to detect the motion singularity (Figure 2), a local property of the optic flow field that would, in some cases, be predictive of the self-motion direction (Gibson, 1950). To explore this, we examined how individual convolutional filters in each model responded to radial and circular optic flow test patterns. Figure 19 shows the responses of 20 exemplar filters from the CNN model to different locations of the four optic flow patterns. A strikingly consistent pattern emerged whereby each filter responds maximally to localized regions at the border of the test patterns. These activations do not coincide with the motion singularity, which appears at the center of the test optic flow patterns. Rather, each filter responds to a preferred local motion direction within the RF, consistent with direction tuning in MT. For example, Neuron 1 (top-left heatmap in Figures 19A–D) prefers –45° planar motion because its maximal activation tracks the position of down-and-rightward motion where ever it appears in the optic flow field—the bottom-right region of the radial expansion pattern (Figure 19A), the top-left of the radial contraction pattern (Figure 19B), etc.

## Discussion

Inspired by the success of accuracy-optimized CNNs at modeling neural properties of the primate ventral stream, we investigated whether neurons in CNNs that are optimized to accurately estimate self-motion effectively model optic flow tuning properties in primate MSTd. While CNN neurons reproduce some characteristics of MSTd neurons, there are important differences. Most model neurons demonstrate peak discriminability for backward headings (Figure 11), whereas the discriminability of forward and backward headings is much more balanced in the MSTd population of Gu et al. (2010). The CNN approximates well the percentages of neurons that have translation preferences around the cardinal axes (Table 4), however, the model exhibits bias toward backward self-motion and horizontal elevations, both of which do not reflect MSTd translation tuning holistically (Figure 9). While the CNN model population yields a heading tuning index that is commensurate to that of MSTd (Figure 12), CNN neurons exhibit greater variability in their tuning widths (Figure 10) than MSTd neurons.

The correspondence between the CNN model variants and MSTd is also inconsistent when it comes to rotational optic flow tuning. The rotational tuning profiles of numerous CNN neurons are multimodal, which does not resemble the unimodal profiles of MSTd neurons (Figure 13). The CNN yields marginal distributions of rotational azimuth and elevation preferences that qualitatively resemble those of the MSTd population, but the model does not reproduce the virtually absent preference for the forward and backward axes of rotation in the MSTd data (Figure 15). Taken together, these findings indicate that the correspondence between the CNN model and primate MSTd is mixed. By contrast, our simulations show that tuning properties emerge within the NNMF model that better align with MSTd in almost every analysis. None of the CNN model variants inspired by NNMF that we examined offer a model that is nearly as compelling as NNMF overall.

### The computational objective of MSTd

What could account for the disparity in how well CNNs and NNMF emulate MSTd optic flow tuning? One major difference is their computational objective—the CNN model and all the variants that we simulated are optimized to accurately estimate the observer's translational and rotational velocity, possibility subject to one or more NNMF-inspired constraints. In contrast, NNMF performs dimensionality reduction for the purpose of reconstructing its motion inputs. Because NNMF imposes a non-negativity constraint, the reconstruction must arise through solely additive combinations of parts of the input signal (Lee and Seung, 1999). In the NNMF model each MT input a MSTd unit receives must exert a positive influence on that MSTd unit's response (Beyeler et al., 2016); none of the MT subunits may contribute inhibition. Even though the CNNs use the ReLU activation to prevent negativity in the neuronal activations, the accuracy-based objective alone does not preclude weights that combine these activations to take on negative values. This allows for the possibility of subtractive interactions (i.e., inhibition), unlike in NNMF. Introducing the non-negative weight constraint within the CNN_++ and CNN_L1++ models results in an additive, parts-based decomposition within the CNN paradigm. Interestingly, this gives rise to more MSTd-like tuning profiles than the other models simulated, particularly in the case of rotation (Figure 13). Given the broader discrepancy between the CNN_++ and NNMF models, our simulations suggest that not every parts-based decomposition is compatible with MSTd optic flow tuning properties. This raises the intriguing possibility that the computational objective of NNMF may be more compatible with that of MSTd than the accuracy-oriented objective of the CNN. This is surprising given that CNNs optimized to accurately classify natural images represent the leading models of neural primate ventral stream (Yamins et al., 2014; Schrimpf et al., 2018; Serre, 2019; Lindsay, 2021).

One possibility for reconciling our findings with those that pertain to the ventral stream is that information could be encoded differently among neurons to best support the overarching computational goals of the dorsal and ventral streams. The ventral stream has long been associated with object recognition (Mishkin et al., 1983; Dicarlo and Cox, 2007) and perhaps the task of accurately classifying natural images on which leading CNN models of the ventral stream are trained align well with the objective subserved by the pathway. Indeed, while CNNs classify images more accurately than humans on the ImageNet natural image dataset (Russakovsky et al., 2015), human misclassification errors are remarkably small, only ≈5% of images. On the other hand, even if self-motion perception is central to the computational objectives of the dorsal stream, many naturalistic scenarios may not demand a readout with veridical accuracy, which could shape the accuracy with which the pathway encodes self-motion from optic flow. While psychophysical studies show that humans are capable of judging their heading from optic flow to within ≈1°, the optic flow displays in these studies consist of pure translation over a limited range (Warren et al., 1988; Crowell and Banks, 1993; Sun et al., 2020)—typically within ≤ 30° of the straight-ahead and yoked to the horizontal axis. These conditions are less complex than even the BenHamedT dataset explored here on which both CNNs and NNMF accurately estimated the simulated self-motion direction. Indeed, errors reach ≈15° when humans judge heading from optic flow over a 360° range in the horizontal plane (Cuturi and Macneilage, 2013). When humans judge heading from optic flow that contains combinations of translation and rotation, errors reach ≈10–15° when heading is only within 6° of the straight-ahead (Royden et al., 1994; Grigo and Lappe, 1999). Taken together, the pattern of human self-motion judgments from optic flow is compatible with a neural representation that does not encode self-motion with near-veridical accuracy.

### Accuracy of self-motion estimation on the TR360 and Ben Hamed datasets

Estimating self-motion from the TR360 optic flow fields used in our study is substantially more challenging than those used in human psychophysical studies. On this dataset, we found that the CNN variants decoded self-motion on the TR360 test set with much higher accuracy than NNMF (Figure 5). This sizable gap in accuracy largely vanished when we tested the models on the BenHamedT and BenHamedR datasets (Figure 6), which have been previously used to evaluate the decoding accuracy of NNMF (Beyeler et al., 2016). The BenHamedT and BenHamedR datasets consist of pure forward translation and pure yaw rotation, respectively, which is far simpler than the complex combinations of translation and rotation that are present in the TR360 dataset. This suggests that while NNMF better captures MSTd optic tuning properties, the model only supports the accurate decoding of self-motion in simplistic novel self-motion scenarios. We find it unlikely that the sparse representations in NNMF alone hinder generalization (Spanne and Jörntell, 2015), since our simulations show that the self-motion estimates obtained from PCA are no more accurate than NNMF, despite not having a sparse representation. It is also possible that NNMF may struggle to encode the complexity of the TR360 dataset with only 64 basis vectors. However, increasing the number of basis vectors to several hundred in our testing did not overcome this issue. Regardless of its basis, the discrepancy in accuracy between the CNN and NNMF models offers a neurophysiological prediction that could help improve our understanding of MSTd. We are not aware of an existing neurophysiological study that reports the accuracy with which translation and rotation may be decoded from MSTd for optic flow stimuli as complex as those in the TR360 dataset. Accurate decoding of self-motion from MSTd would be consistent with the accuracy-driven mechanisms in the CNN, whereas poor accuracy would further strengthen the case for the NNMF model.

### Nonnegativity and sparseness constraints

What is the computational goal of MSTd if it is not accurate self-motion estimation? Beyeler et al. (2019) argue that nonnegativity and sparseness represent computational principles that give rise to a wide range of neural properties, including optic flow tuning in MSTd. The CNN variants that incorporate these computational constraints through nonnegative weights and L1 regularization (CNN_L1, CNN_++, CNN_L1++) do not produce more effective models of optic flow tuning in MSTd than NNMF. This suggests that these computational constraints are not sufficient for capturing optic flow tuning in MSTd. Given that the CNN_L1 and CNN_L1++ models have higher proportions of unresponsive neurons compared to the other CNNs (Figure 18), the L1 regularization in these models appears to be promoting the expected sparseness effect. Despite this, it is noteworthy that the hyperparameters of these models were selected to achieve the highest self-motion estimation accuracy. This does not rule out the possibility that modifying the CNN cost function to prioritize sparseness over self-motion motion accuracy may produce more MSTd-like tuning (Kashyap et al., 2019; Layton and Fajen, 2022). However, even when the hyperparameter search process yields networks with sparser representations, as is the case for MLP (Figure 16), they do not produce optic flow tuning that is better aligned with MSTd.

We find that the non-negativity weight constraint alone does not consistently yield more MSTd-like tuning across most measures. The translation direction preferences of neurons in the CNNs with the non-negativity constraint (CNN_++, CNN_L1++) are markedly different from MSTd (Figure 9). The CNN_L1++ model that implements both sparseness and non-negativity constraints does not approximate the translation and rotation tuning characteristics of MSTd better than the CNN_++ model that incorporates only the non-negativity constraint. This suggests that the two factors may not act synergistically. It is, however, worth noting the nonnegativity constraint may encourage unimodal and structured translation and rotation tuning profiles that are more similar to NNMF than the other networks which have multimodal, scattershot profiles (Figures 8, 14).

### Convolution and encoding of optic flow

We simulated MLPs to better understand the potential role of convolution on the encoding of optic flow. Recall that the MLPs possess the same architecture as the CNNs, except they lack the convolution stack early in the neural network (Figure 4A). While the MLP models do not differ substantially from the CNNs with respect to the accuracy of self-motion estimates, many MLP neurons demonstrate exceptional selectivity to certain translational optic flow patterns. This is evident from the subpopulation of neurons with HTI values close to 1.0 (Figure 12) and the emergence of “point” tuning profiles wherein a neuron only responds to a single optic flow pattern (e.g., Figure 7; MLP Neurons 2 and 3), analogous to grandmother cell selectivity. By contrast, the CNN, CNN_++, and CNN_L1++ models produce broader, more extended tuning profiles. It is noteworthy that this pattern emerged in both the MLP and MLP_MT_PRE models that were optimized independently, which suggests that the fully connected pattern of connectivity promotes localist coding. Interestingly, NNMF implements the same kind of dense connectivity, yet it yields entirely different tuning characteristics.

Recall that the MLP and MLP_MT_PRE models differ in the format of their motion inputs — the CNNs and the MLP model both process optic flow whereas MLP_MT_PRE processes the same MT-like motion signal that NNMF does. Across our analyses, it is remarkable that these two motion input representations made little difference in the resulting optic flow tuning.

### Additional mechanisms and training protocol

The insufficiency of the CNNs, even with NNMF-inspired constraints, may result from the omission of an important yet-to-be-identified mechanism. Maus and Layton (2022) found that accuracy-optimized CNNs do not match the accuracy of human heading perception from optic flow in important scenarios including in the presence of independently moving objects, scenes that produce sparse optic flow fields, and in the presence of visual rotation. Processing optic flow over time with recurrent connections improved the consistency with human self-motion judgments, so the inclusion of this mechanism might also yield more MSTd-like tuning properties. It is noteworthy, however, that NNMF achieves much better consistency with MSTd than the CNNs without processing optic flow over time.

Alternatively, the discrepancy between the CNNs and MSTd may stem from assumptions within the selected deep learning framework. Training CNNs on a dataset with a large number of labels using a supervised learning paradigm may align well with the computational objectives of ventral stream (Khaligh-Razavi and Kriegeskorte, 2014), but perhaps not those of the dorsal stream. For example, it has been argued that the self-supervised learning paradigm represents a more biologically plausible learning method since it relies on stimulus-derived predictions to drive learning in lieu of ground truth labels (Rao and Ballard, 1999; Mineault et al., 2021; Halvagal and Zenke, 2023). Another possibility is that supervised learning based neural networks may be compatible with the dorsal stream, but the goal is something other than achieving accurate self-motion estimation, as we assumed here. For example, MSTd may participate in a larger system that optimizes perception-action objectives that subserve successful dynamic interactions with the environment, such as navigation toward goals (Page et al., 2015; Page and Duffy, 2018; Alefantis et al., 2022).

## Conclusion

Our findings indicate that CNNs that are optimized to accurately estimate the observer's translational and rotational self-motion from optic flow do not effectively capture the collection of MSTd optic flow tuning properties examined here. The NNMF model that performs dimensionality reduction on its motion inputs resulted in tuning that more consistently emulates MSTd tuning characteristics. Incorporating a sparseness constraint inspired by NNMF into the CNNs did not produce more compelling models of MSTd overall, while incorporating a nonnegativity constraint did improve correspondence with MSTd across some measures (e.g., rotational tuning). We conclude that additional architectural constraints or computational goals beyond sparsity and nonnegativity are necessary for the emergence of MSTd-like tuning characteristics.

## Data Availability

The datasets presented in this study can be found in online repositories. The names of the repository/repositories and accession number(s) can be found in the article/Supplementary material.
