# Supplementary material for: Accuracy optimized neural networks do not effectively model optic flow tuning in brain area MSTd
Source: Front Neurosci. 2024 Sep 2;18:1441285. doi: 10.3389/fnins.2024.1441285 (PMC11403719; doi:10.3389/fnins.2024.1441285)
Supplement: Supplementary file 1 [file Data_Sheet_1.pdf]

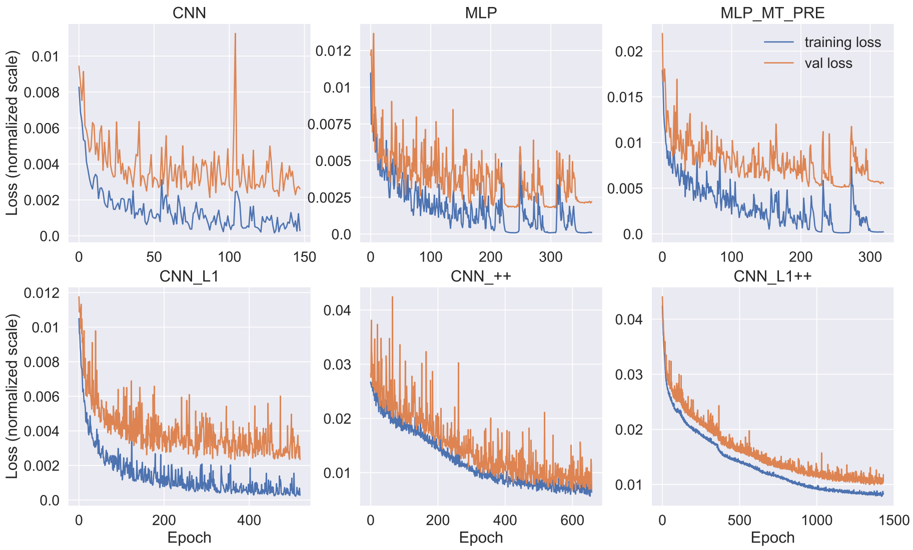

Figure S1: Loss achieved on the training and validation sets during training of the neural network models indicated. The x-axis corresponds to the training epoch and y-axis corresponds to the loss achieved on the normalized self-motion labels (see Materials and Methods). The loss over the first few epochs are omitted from the plots to focus on the long-term structure.
